# The Expanding Role of Non-Coding RNAs in Neurodegenerative Diseases: From Biomarkers to Therapeutic Targets

**DOI:** 10.3390/ph19010092

**Published:** 2026-01-03

**Authors:** Xuezhi Zhao, Yongquan Zheng, Xiaoyu Cai, Yao Yao, Dongxu Qin

**Affiliations:** 1 Department of Gynecology, Women’s Hospital, School of Medicine, Zhejiang University, Hangzhou 310006, China; zhaoxz0802@zju.edu.cn; 2 Department of Pharmacy, Women’s Hospital, School of Medicine, Zhejiang University, No. 1 Xueshi Road, Shangcheng District, Hangzhou 310006, China; 5515058@zju.edu.cn; 3 Department of Pharmacy, Affiliated Hangzhou First People’s Hospital, School of Medicine, Westlake University, Hangzhou 310006, China; 5519066@zju.edu.cn

**Keywords:** non-coding RNAs, neurodegenerative diseases, Alzheimer’s disease, Parkinson’s disease, RNA biomarkers, RNA therapeutics

## Abstract

Non-coding RNAs have emerged as central regulators of gene expression in neurodegenerative diseases, offering new opportunities for diagnosis and therapy. This review synthesizes current knowledge on microRNAs, long non-coding RNAs, and circular RNAs in Alzheimer’s disease, Parkinson’s disease, and amyotrophic lateral sclerosis, emphasizing their roles in synaptic function, proteostasis, mitochondrial biology, and neuroinflammation. We evaluate evidence supporting non-coding RNAs as circulating and tissue-based biomarkers for early detection, disease monitoring, and patient stratification, and we compare analytical platforms and biofluid sources. Mechanistic insights reveal how non-coding RNAs modulate pathogenic protein aggregation, neuronal excitability, immune cell crosstalk, and blood–brain barrier integrity. Translational efforts toward RNA-targeted interventions are reviewed, including antisense oligonucleotides, small interfering RNAs, miRNA mimics and inhibitors, circular RNA decoys, and extracellular vesicle-mediated delivery systems. We discuss pharmacological modulation, delivery challenges, safety concerns, and strategies to enhance specificity and CNS penetration. Finally, we outline emerging computational and multi-omics approaches to prioritize therapeutic targets and propose a roadmap for advancing non-coding RNA research from preclinical models to clinical trials. Addressing biological heterogeneity and delivery barriers will be pivotal to realizing the diagnostic and therapeutic promise of the non-coding transcriptome in neurodegenerative disease. Collaboration across disciplines and rigorous clinical validation are urgently needed.

## 1. Introduction

As populations age globally, neurodegenerative diseases pose a major challenge to public health worldwide [1]. These disorders, including Alzheimer’s disease (AD) and Parkinson’s disease (PD), currently affect over 57 million individuals globally, with estimates projecting the doubling of cases in the next two decades [2]. Characterized by progressive neuronal loss in the central nervous system, they are associated with high disability and mortality rates [3]. Nevertheless, their highly heterogeneous etiologies, extended preclinical phases, and incompletely understood pathogenesis make early diagnosis exceedingly difficult [4]. Currently, no effective disease-modifying treatments exist in clinical settings, where available therapies merely alleviate symptoms [5]. This urgent need has shifted research focus to molecular mechanisms for identifying early diagnostic biomarkers and therapeutic targets [6]. Recently, RNA regulatory pathways have attracted considerable attention, positioning non-coding RNA (ncRNA) as a key focus in neurodegenerative disease studies owing to its central role in gene expression regulation [3]. Previously regarded as “transcriptional noise,” ncRNA is now recognized as an essential regulator of gene expression, with critical functions in neural development and disease etiology [7].

ncRNAs encompass a diverse class of RNA molecules that do not code for proteins but modulate gene function at transcriptional, post-transcriptional, and epigenetic levels [8]. Subtypes include microRNAs (miRNAs), long non-coding RNAs (lncRNAs), circular RNAs (circRNAs), and PIWI-interacting RNAs (piRNAs) [9]. miRNAs, typically ~22 nucleotides (nt) long, mediate post-transcriptional gene silencing by base-pairing with the 3′ untranslated region of target mRNAs [10]. lncRNAs (>200 nt) act as scaffolds for chromatin-remodeling complexes in the nucleus to regulate transcription or, in the cytoplasm, serve as molecular sponges that sequester proteins or miRNAs, thereby influencing mRNA stability and translation [5]. circRNAs form via back-splicing of pre-mRNA, yielding stable closed loops lacking 5′ and 3′ ends; they are enriched in neural tissues and often function as sponges for miRNAs or RNA-binding proteins to control gene expression [10]. piRNAs (24–32 nt) were first discovered in germ cells, where they guide PIWI complexes to induce histone methylation or cleave transposon transcripts, silencing transposons and maintaining genomic integrity [11]. Emerging evidence indicates their expression in mammalian neurons, potentially regulating functions like learning and memory [8,11]. Many ncRNAs exhibit tissue- and stage-specific expression in the nervous system; for example, numerous lncRNAs show high, region- and development-specific expression in the brain [8], circRNAs accumulate in aging neurons [10], and the piRNA–PIWI pathway suppresses transposons in adult neurons beyond germ cells [11].

**Figure 1:**
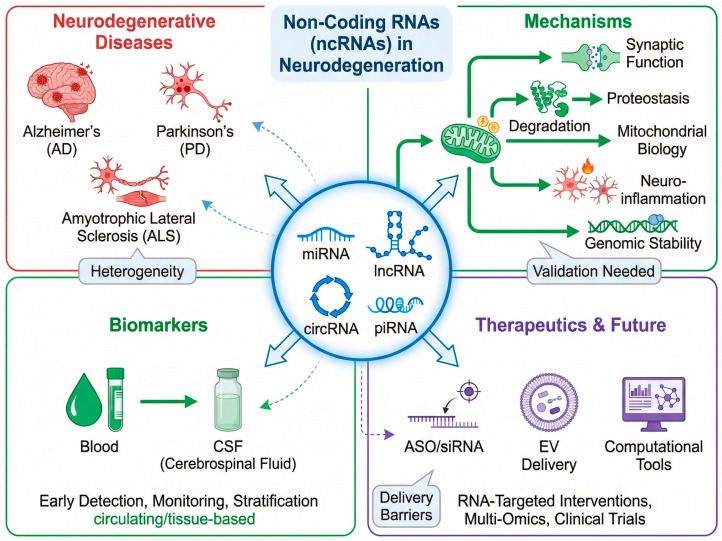
The Expanding Role of Non-Coding RNAs in Neurodegenerative Diseases: From Biomarkers to Therapeutic Targets.

Accumulating evidence reveals that ncRNA dysregulation underlies key pathological processes in neurodegenerative diseases [3] (Figure 1). Dysregulated ncRNA expression or function can compromise synaptic plasticity, destabilize neural network homeostasis, and alter pathogenic protein dynamics—via transcriptional and translational control—ultimately disrupting proteostasis [6]. Additionally, such dysregulation modulates cerebral immune responses molecularly, intensifying chronic neuroinflammation [12]. Of note, impairments in pathways like piRNA/PIWI-mediated transposon silencing may derepress transposons, escalate genomic instability, and cause progressive neuronal injury [7]. These findings highlight ncRNA’s pivotal roles in synaptic regulation, proteostasis, inflammation, and genomic stability, providing fresh insights into disease pathogenesis and therapeutic innovation [4].

Since the foundational reviews by Rege et al. (2013) [13] and recent updates by Mo (2023) [14], several transformative advances warrant renewed synthesis: (1) the clinical translation of ncRNA-targeting therapeutics, exemplified by FDA-approved tofersen for SOD1-ALS (2023); (2) recognition of epitranscriptomic regulation (m6A, A-to-I editing) as a critical modifier of ncRNA function; (3) emergence of single-cell and spatial transcriptomics enabling cell type-specific ncRNA profiling; and (4) accumulating evidence—both positive and negative—from clinical trials that inform future therapeutic development. Given ncRNA’s crucial role in neurodegenerative disease pathogenesis, this review employs a ‘mechanism–evidence level–translational path’ framework to systematically evaluate recent progress in the field. Unlike prior reviews that predominantly catalog ncRNA expression changes [13,14], our synthesis critically evaluates evidence quality by distinguishing preclinical from clinical findings and animal models from human studies; integrates the emerging crosstalk between ncRNAs, RNA-binding proteins, and epitranscriptomic modifications—a regulatory layer frequently overlooked in ncRNA-centric summaries; and provides critical appraisal of clinical trial outcomes, including failures such as the tominersen phase 3 futility, to extract mechanistic lessons for future therapeutic development. This review covers literature published between 2011 and May 2025, with emphasis on studies from 2020–2025. It aims to elucidate ncRNA mechanisms, assess the scope and rigor of current evidence, and explore translational avenues to clinical practice, offering guidance for future research and interventions.

## 2. The Biological Basis of ncRNAs in the Nervous System

### 2.1. miRNA and the Nervous System

**Figure 2:**
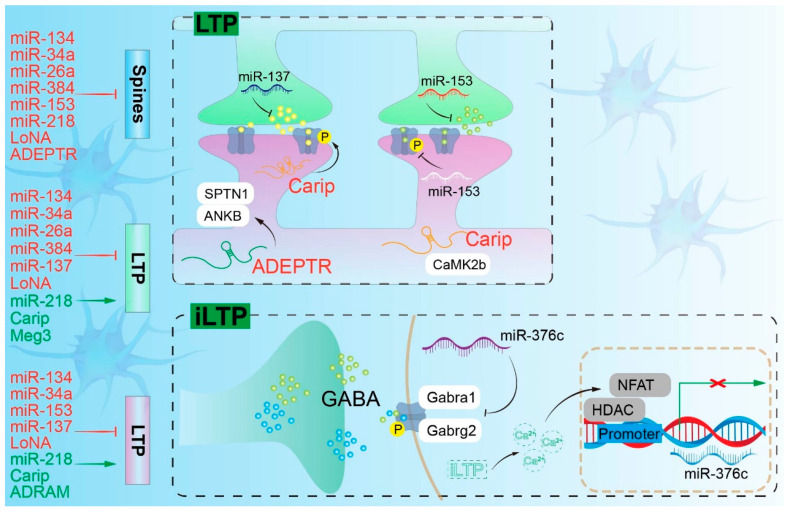
Roles of non-coding RNAs in the remodeling of neuronal circuits. Representative ncRNAs regulating LTP and memory formation. The schematic overview on the left shows individual miRNAs and lncRNAs that promote (green) or inhibit (red) dendritic spine formation, long-term potentiation (LTP), or memory formation. The reported mechanistic interactions between ncRNAs and their targets are further illustrated within the relevant cellular compartments. During inhibitory long-term potentiation (iLTP), expression of miR-376c is downregulated, thereby allowing translation of Gabra1 and Gabrg2, both of which encode subunits of inhibitory receptors. ADEPTR, activity-dependent lncRNA ADEPTR; ADRAM, Activity-Dependent lncRNA Associated with Memory; ANKB, Ankyrin-B; Ca^2+^, Calcium ion; CaMK2b, Calcium/Calmodulin-Dependent Protein Kinase II beta; Carip, CaMKIIb-associated lncRNA captured via immunoprecipitation; GABA, γ-Aminobutyric Acid; Gabra1, GABA(_A) receptor subunit alpha1; Gabrg2, GABA(_A) receptor subunit gamma2; HDAC, Histone Deacetylase; iLTP, inhibitory Long-Term Potentiation; LoNA, nucleolus-localized lncRNA LoNA; LTP, Long-Term Potentiation; Meg3, Maternally Expressed Gene 3; NFAT, Nuclear Factor of Activated T-cells; P, Phosphorylation; SPTN1, Spectrin Alpha Chain, Non-Erythrocytic 1.

**Figure 3:**
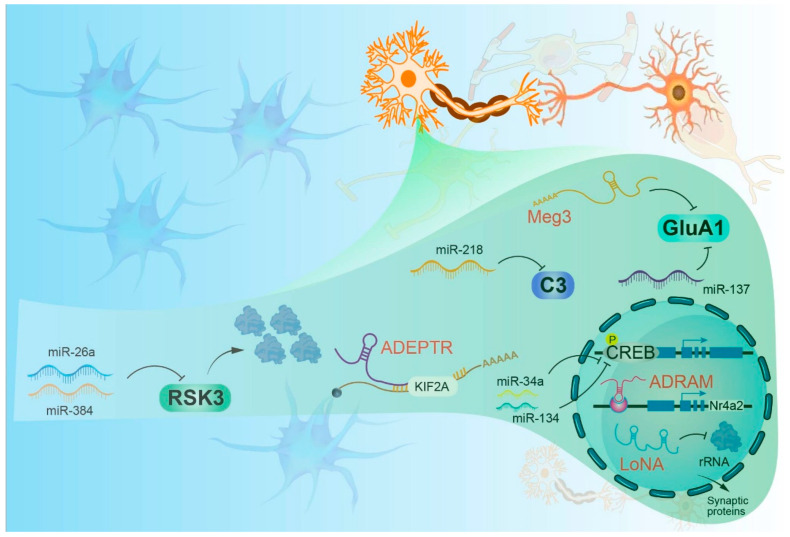
Non-coding RNA–mediated regulation of neuronal activity-dependent synaptic pathways. Schematic illustration of representative microRNAs (miRNAs) and long non-coding RNAs (lncRNAs) that modulate synaptic composition and plasticity through post-transcriptional and transcriptional mechanisms. In the cytoplasm, miR-26a/miR-384 regulate RSK3 signaling, the activity-dependent lncRNA ADEPTR is linked to KIF2A-associated transport, and miR-218 targets complement C3; in parallel, Meg3 and miR-137 influence the AMPA receptor subunit GluA1. In the nucleus, miR-34a/miR-134 tune CREB activation, the activity-dependent lncRNA ADRAM supports CREB-associated transcription of Nr4a2, and the nucleolus-associated lncRNA LoNA regulates rRNA biogenesis and downstream synaptic protein synthesis. Arrows denote reported regulatory relationships. ADEPTR, Activity-regulated synaptic targeting of lncRNA ADEPTR; ADRAM, Activity-dependent lncRNA associated with memory; C3, Complement Component 3; CREB, cyclic adenosine monophosphate (cAMP) Response Element Binding Protein; GluA1, Glutamate receptor subunit A1 (AMPA receptor subunit 1); KIF2A, Kinesin Family Member 2A; LoNA, Long nucleolus-associated RNA (nucleolus-localized lncRNA); Meg3, Maternally Expressed Gene 3; Nr4a2, Nuclear Receptor Subfamily 4 Group A Member 2; P, Phosphorylation (phosphate group); rRNA, ribosomal RNA; RSK3, Ribosomal S6 Kinase 3.

miRNAs are approximately 22 nucleotides long and mediate post-transcriptional gene silencing by binding to target mRNAs, leading to mRNA degradation or translational repression. Their biogenesis entails the nuclear processing of primary miRNAs (pri-miRNAs) into precursor miRNAs (pre-miRNAs) by Drosha and DGCR8, followed by cytoplasmic cleavage by Dicer to produce mature miRNAs, which are then incorporated into the RNA-induced silencing complex (RISC) [15,16]. In neurons, this pathway is crucial for differentiation and survival, with its dysregulation linked to impaired maturation and synaptic dysfunction. For instance, NMDA receptor activation modulates the levels of DGCR8 and Drosha, thereby affecting miRNA processing and synaptic plasticity [17]. Neuron-specific conditional knockout of Dicer leads to neurodegenerative alterations, highlighting the role of biogenesis defects in disorders such as Rett syndrome [18,19]. miRNAs also localize to axons and dendrites, where they regulate local translation—a process essential for activity-dependent plasticity [20]. miR-134 regulates dendritic spine morphology by repressing Limk1; its dysregulation impairs long-term potentiation (LTP) in fragile X syndrome [21,22]. In pain models, miRNAs modulate neuronal excitability and synaptic strength [23]. miRNAs also contribute to homeostatic plasticity by maintaining network balance via synaptic scaling in response to chronic activity changes [23,24,25,26]. In inflammatory contexts, miRNAs target pro-inflammatory pathways; for example, miR-146a suppresses NF-κB signaling in microglia, thereby mitigating neuroinflammation in amyotrophic lateral sclerosis (ALS) and PD, while its downregulation enhances cytokine responses and aggravates neurodegeneration [27,28,29]. Regarding proteostasis, miRNAs such as miR-153 target amyloid precursor protein (*APP*) in AD to preserve protein homeostasis [30]; their depletion promotes aberrant tau phosphorylation and aggregation, thereby driving tau pathology [31]. Collectively, miRNAs serve as dual regulators of neuroprotection and neuropathology, demonstrating therapeutic potential via the modulation of synaptic and inflammatory networks. As summarized in Figure 2 and Figure 3, a broad repertoire of miRNAs and lncRNAs orchestrates dendritic spine dynamics, long-term potentiation, and memory by either promoting or constraining synaptic strength across distinct subcellular compartments, with miR-376c-mediated control of Gabra1 and Gabrg2 during iLTP providing a representative example of inhibitory circuit remodeling (Figure 2 and Figure 3).

### 2.2. lncRNAs and the Nervous System

**Figure 4:**
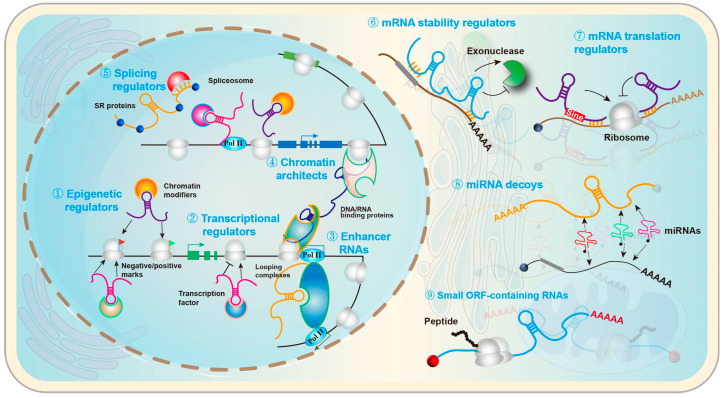
Regulatory roles of lncRNAs within the cell.

lncRNAs, typically >200 nucleotides, regulate gene expression through chromatin remodeling, antisense-mediated interference, and competing endogenous RNA (ceRNA) mechanisms, among others [32,33]. As a paradigm, NEAT1 serves as an architectural scaffold for nuclear paraspeckles, whose assembly and function rely on NEAT1’s modular domains [34]. Under proteotoxic stress, the heat-shock transcription factor HSF1 binds a conserved heat-shock element in the NEAT1 promoter, upregulating NEAT1 and driving paraspeckle formation as part of the cellular heat-shock response [35]. In Huntington’s disease (HD), NEAT1 levels are altered in patient tissue and models, and experimental manipulation suggests a neuroprotective role under oxidative stress; huntingtin itself binds NEAT1 and localizes to paraspeckles, linking paraspeckle biology to HD pathogenesis [36,37,38]. NEAT1 is frequently dysregulated across cancers and neurodegenerative disorders, consistent with its central role in stress responses and nuclear organization [38,39,40]. In neurodegeneration, NEAT1/paraspeckles rise during heat-shock and intersect with molecular programs implicated in Alzheimer’s and Parkinson’s disease. For example, NEAT1 modulates inflammatory and survival pathways in PD models via a NEAT1–miR-124 axis, illustrating how lncRNAs integrate transcriptional control with miRNA “sponging” to shape neuroinflammation and disease course [40,41]. Antisense lncRNAs also act as feed-forward amplifiers of pathology. BACE1-AS stabilizes BACE1 mRNA and functions as a ceRNA to counter miRNA-mediated repression, thereby increasing β-secretase activity and enhancing Aβ production in AD models and patient samples [42,43]. Within ceRNA networks in the brain, lncRNAs can facilitate neuronal differentiation by sequestering lineage-regulatory miRNAs. The primate lncRNA LncND, for instance, sponges miR-143-3p to tune Notch signaling in neural progenitors, promoting proper neurodevelopmental trajectories [44]. Conversely, when stress-responsive RNA condensates are disrupted, toxicity can worsen: NEAT1 promotes formation of protective TDP-43 nuclear bodies via liquid–liquid phase separation; perturbation of this pathway and TDP-43/paraspeckle cross-talk is linked to ALS pathogenesis [45,46]. In AD, NEAT1 upregulation has been tied to redox and tau pathways: NEAT1 influences microtubule stability through the FZD3/GSK3β/p-tau axis and modulates oxidative-stress readouts in cellular and mouse models—findings that together suggest a context-dependent, potentially adaptive stress response with therapeutic implications [47,48]. Figure 4 provides an overview of the diverse regulatory modes by which lncRNAs act in neurons, including chromatin scaffolding, transcriptional control, modulation of RNA processing and stability, and ceRNA-like interactions with miRNAs and RBPs that together rewire gene-expression programs in neurodegeneration (Figure 4).

### 2.3. circRNAs and the Nervous System

circRNAs arise through back-splicing [49]. Because they resist exonucleolytic degradation, circRNAs are highly stable and abundant in the brain [50,51]. They also progressively accumulate with age in post-mitotic neurons [52,53]. This pronounced stability yields higher steady-state levels than those of other ncRNAs [50,54]. circRNAs regulate gene expression by acting as miRNA sponges [55], thereby influencing transcription and translation [54]. In the central nervous system (CNS), circRNAs are dynamically expressed and act via miRNA sponging and protein interactions. Their stability enables sustained regulation of gene networks across diverse neurological contexts [51,54]. miRNA sponging is widely considered a principal mechanism: circRNAs function as decoys that block miRNA-mediated repression of target mRNAs, thereby influencing neurodevelopment and disease [55,56,57]. In parallel, circRNA–protein interactions allow circRNAs to scaffold molecular complexes and modulate synaptic function [54,57,58]. Their age-related accumulation in neurons further suggests roles in memory and other cognitive processes [59,60]. Taken together, circRNAs represent promising biomarkers and therapeutic targets for CNS disorders, with their stability providing a basis for durable regulatory effects.

### 2.4. piRNAs and the Nervous System

piRNAs are 24–32-nt noncoding RNAs that associate with PIWI proteins to exert epigenetic regulation and suppress activation of transposable elements [61,62]. By targeting transposon-derived RNAs, piRNAs induce degradation or transcriptional silencing, thereby preventing genomic insertion and preserving genome stability [61,62]. In adult neurons, piRNA–PIWI complexes maintain transposon silencing primarily through heterochromatin formation, thereby safeguarding genomic integrity [63,64]. In neurodegenerative disorders—particularly tauopathies (e.g., AD and PD)—piRNA dysregulation can lead to aberrant activation of transposable elements, which promotes heterochromatin decondensation, facilitates genomic insertion of transposons, disrupts genome stability, and precipitates neuronal death and inflammatory responses [65,66,67]. These events not only inflict genomic damage but may also provoke immune activation, thereby accelerating disease progression [67,68]. Evidence also indicates piRNA dysregulation in ALS, suggesting that this pathway could serve as a biomarker for neurodegenerative disease [69,70]. Critically, piRNA research in neurodegeneration remains at an early stage compared to miRNA studies: most findings derive from Drosophila models or postmortem human tissue, with limited longitudinal or functional validation; the specific piRNA species involved differ substantially across studies, and standardized profiling methods are lacking. Beyond transposon control, piRNAs also contribute to neuronal development, axonal regeneration, and memory formation; by restraining transposon-mediated inflammatory responses, they help maintain genome stability in the aging brain and modulate nervous system function [11]. Consequently, piRNAs are pivotal for neural homeostasis and may offer novel targets for early diagnosis and therapy of neurodegenerative disorders.

### 2.5. RNA-Binding Proteins (RBPs) and Epitranscriptome

**Figure 5:**
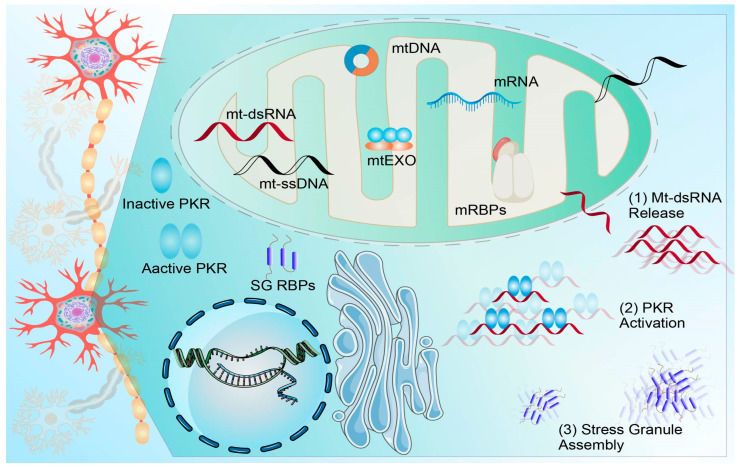
Key links among RNA, mitochondria, and the stress response in neurodegeneration. Inside cells there is a highly sensitive defense program known as the integrated stress response (ISR). It acts like an emergency braking system in a machine: once danger signals are detected, it rapidly slows protein synthesis to prevent further damage. Activation: four “stress-sensing” kinases are engaged under distinct conditions: HRI responds to oxidative stress; PERK responds to misfolded proteins in the endoplasmic reticulum; GCN2 responds to nutrient deprivation; and PKR responds to the accumulation of double-stranded RNA. These kinases phosphorylate the initiation factor eIF2α, transiently halting global protein synthesis. At the same time, cellular RNAs and proteins are condensed into specialized stress granules, where they are stored until the threat has passed and translation can resume. Under normal conditions, this response is short-lived, acting like a brief protective “booster.” However, if the ISR remains chronically activated, neurons become trapped in a state of energy shortage and functional impairment, ultimately progressing toward degeneration. Aging is not driven by a single cause, but instead emerges from the convergence of multiple forms of cellular stress, and RNA itself may serve as a “seed” of chronic stress. Understanding and targeting this process may not only help delay aging, but also open new avenues for combating intractable disorders such as Alzheimer’s disease and Parkinson’s disease: RNA is not only a carrier of information but also a hidden driver of neuronal aging.

RBPs, such as TDP-43 and FUS, are central regulators of RNA homeostasis in neurodegeneration. They cooperate with Drosha and Dicer to drive miRNA biogenesis, while pathogenic mutations, nucleocytoplasmic transport defects, or aberrant liquid–liquid phase separation (LLPS) disrupt this process, causing miRNA dysregulation in ALS and increasing motor-neuron vulnerability [71,72,73]. In parallel, epitranscriptomic modifications—most notably m6A methylation and A-to-I editing—remodel post-transcriptional fates by modulating splicing, localization, translation, and decay; among them, the METTL3/14–YTH axis and ADAR-mediated editing are particularly critical during brain aging and stress [74,75]. TDP-43 and FUS associate with distinct mRNA networks, thereby dictating mRNA stability and local translation within axons and dendrites. When these RBPs aggregate into stress granules and sequester processing factors, the m6A landscape is concomitantly reconfigured, establishing a vicious cycle that accelerates neuronal loss [76]. Accordingly, precise interventions targeting RBP phase separation, nucleocytoplasmic transport, and epitranscriptomic writers, erasers, and readers—such as antisense oligonucleotides [77], ADAR-directed editing, or modulation of the m6A pathway—represent promising strategies for treating aging-related neurological diseases. Together, these pathways converge on the integrated stress response, in which ISR kinases, eIF2α phosphorylation, stress granule assembly, and mitochondrial dysfunction couple diverse cellular insults to chronic RNA-mediated toxicity and progressive neuronal loss (Figure 5).

## 3. ncRNAs in Specific Neurodegenerative Diseases

### 3.1. ncRNAs in AD: Regulation of Amyloid-Beta Production, Tau Pathology, and Synaptic Dysfunction

In AD, ncRNAs mediate post-transcriptional regulation that interconnects Aβ production, tau pathology, and synaptic dysfunction into a mutually reinforcing network. Along the Aβ-production axis, the miR-29 family binds the 3′ untranslated region (3′UTR) of BACE1 mRNA to suppress its translation, thereby limiting β-secretase levels and reducing APP cleavage into Aβ peptides (validated by luciferase reporter assays in cell lines). In AD brain tissue [human postmortem studies, *n* = 20–50], miR-29a/b downregulation elevates BACE1, promotes Aβ accumulation, and drives plaque formation; in sporadic AD samples, miR-29 expression inversely correlates with BACE1 activity. This pathway also features feedback regulation: accumulating Aβ further suppresses miR-29 expression, creating a self-reinforcing vicious cycle (demonstrated in Aβ-treated cell cultures). While the miR-29–BACE1 axis has robust mechanistic support from cell and rodent studies, the causal role of miR-29 downregulation in human AD progression remains unproven—human data are correlative and cross-sectional, and no clinical trials have tested miR-29 restoration in AD patients, representing a critical translational gap [78,79,80]. Acting as an “amplifier,” the long noncoding RNA BACE1-AS forms a duplex with BACE1 mRNA to enhance its stability and increase β-secretase activity; it is frequently upregulated in AD, thereby sustaining a positive feedback loop with Aβ [42,78]. Along the tau–synapse axis, miR-132 targets genes such as PTEN and FOXO3 to modulate AKT signaling, thereby influencing neuronal survival, synaptic plasticity, and tau hyperphosphorylation [81,82,83]. In the AD hippocampus, reduced miR-132 is associated with worsened tau pathology and cognitive decline, whereas restoring miR-132 in animal models rescues synaptic or neurogenic deficits [42,82]. Conversely, miR-34c upregulation represses targets linked to survival pathways (e.g., SIRT1) and synaptic function (e.g., SYT1), promoting apoptosis/mitochondrial stress and contributing to neuronal loss and memory impairment [83,84]. At the translational interface, exosome-encapsulated ncRNAs constitute an accessible source of fluid biomarkers. Altered levels of neuron-derived exosomal miRNAs—such as miR-29 family members and miR-135a—in cerebrospinal fluid (CSF) or plasma are used to track disease progression via multi-analyte panels that combine exosomal miRNAs (e.g., miR-29a, miR-125b) improve diagnostic accuracy, with reported sensitivities exceeding 80% in cohort studies [85,86,87]. When these circulating ncRNA features are integrated with imaging via machine-learning approaches, multimodal models further enhance noninvasive early detection and conversion prediction performance [88,89]. In sum, ncRNAs span and interlink the Aβ and tau pathological modules. They participate directly in pathogenesis while offering actionable targets, and miRNA mimics or antisense inhibitors directed at specific molecules represent promising therapeutic avenues. However, critical evaluation reveals important caveats: most miRNA–target relationships have been validated only in cell lines or rodent models, with limited confirmation in human brain tissue; expression changes are often modest (1.5–2-fold), raising questions about pathogenic significance versus epiphenomenal associations; and the direction of causality—whether ncRNA dysregulation drives pathology or results from it—remains unresolved for most candidates [90]. Future studies must prioritize mechanistic validation in human iPSC-derived neurons and integrate ncRNA profiling with genetic perturbation approaches to establish causal relationships.

### 3.2. ncRNAs in PD: Modulation of Alpha-Synuclein Aggregation, Autophagy-Lysosomal Pathways, and Mitochondrial Homeostasis

ncRNAs permeate the pathogenic cascade of Parkinson’s disease (PD), modulating α-synuclein (SNCA) aggregation, the autophagy–lysosomal system, and mitochondrial homeostasis, ultimately culminating in dopaminergic neuron loss [91,92]. Within the miR-7–SNCA–autophagy/mitochondria axis, miR-7 binds the 3′UTR of SNCA mRNA to repress translation, thereby limiting Lewy body–like aggregation [93]. In PD, oxidative stress–driven downregulation of miR-7 induces SNCA overexpression and misfolding and, by impairing mitochondrial function (including complex I activity), further exacerbates mitochondrial dysfunction [92,93]. In parallel, miR-7 is linked to autophagy regulation and facilitates SNCA clearance, while lysosomal–autophagic pathways—centered on LAMP2A–mediated chaperone-mediated autophagy (CMA)—are crucial for α-synuclein degradation; disruption of this axis aggravates proteotoxic stress [94]. Upstream, circRNAs such as circSNCA sponge miR-7, reducing its availability and indirectly increasing SNCA expression; in experimental models, circSNCA elevation is associated with autophagy suppression and redox stress, whereas the antiparkinsonian agent pramipexole downregulates circSNCA, restores miR-7, and attenuates apoptosis, indicating that pharmacological recalibration is achievable at the ncRNA level [95,96]. At the level of mitochondrial quality control, miR-34b/c targets DJ-1 and Parkin—key regulators of mitophagy—and its dysregulation compromises quality control and produces energetic deficits in substantia nigra neurons [97]. As fluid biomarkers, neuron-derived extracellular vehicles (EVs) from cerebrospinal fluid or serum carry miRNAs including miR-7, miR-153, and miR-19b; altered levels of these miRNAs correlate with PD severity and α-syn pathology, and panels integrating EV-miRNAs with circRNAs show promising early diagnostic performance in longitudinal or cohort studies [98,99,100,101]. EVs thus provide a noninvasive window into brain pathology, with miR-7-related EV signatures indicating stress within the dopaminergic system [99,100]. Therapeutically, ncRNA-based strategies are emerging; for example, augmenting miR-7 reduces SNCA burden, supports autophagy, and mitigates α-syn fibril–induced neurodegeneration in vivo (rodent MPTP and A53T transgenic models), outlining a precision pathway—restoring the miR-7 axis, reducing SNCA burden, and correcting autophagy/mitochondrial imbalance—with translational promise. However, systematic evaluation reveals significant translational hurdles: (1) miR-7 efficacy has been demonstrated only in toxin-induced or transgenic rodent models that do not fully recapitulate progressive human PD; (2) human postmortem evidence for miR-7 downregulation in PD substantia nigra is limited (*n* < 100 across 3 studies) and confounded by dopaminergic cell loss; (3) no clinical trials have tested miR-7 augmentation, and optimal CNS delivery methods remain undefined; (4) long-term safety of chronic SNCA suppression is unknown given α-synuclein’s physiological synaptic functions. Together with upstream circRNA modulation and EV-based monitoring, this framework links pathogenesis to diagnostic–therapeutic evaluation [93,102]. Representative miRNAs and lncRNAs with experimentally validated mechanistic roles in the pathogenesis of Alzheimer’s disease and Parkinson’s disease are summarized in Table 1.

### 3.3. ncRNAs in HD: RNA Toxicity from CAG Repeats, Splicing Dysregulation, and RNA-Targeted Therapeutic Interventions

HD results from abnormal expansion of the CAG repeat in the HTT gene [106]. Beyond the toxicity of mutant huntingtin (mHTT), expanded CAG transcripts readily form hairpin structures that sequester RNA-binding proteins (e.g., MBNL1), thereby perturbing pre-mRNA splicing and miRNA biogenesis and driving widespread transcriptomic remodeling [107,108,109]. This RNA toxicity also triggers nucleolar dysfunction and ribotoxic stress and, to a considerable degree, occurs independently of mutant protein aggregates [110,111]. Accordingly, RNA-targeted interventions have emerged as a major therapeutic strategy. First, antisense oligonucleotides (ASOs, e.g., tominersen/RG6042) reduce HTT concentrations in CSF; however, their lack of allele specificity has led to inconsistent clinical outcomes [112,113]. Second, nanoparticle-delivered siRNAs designed around SNPs linked to the mutant allele selectively silence mutant HTT while preserving wild-type function in preclinical models [113,114]. In addition, adeno-associated virus (AAV) vectors expressing artificial miRNAs (AAV-miRNAs) embed an siRNA within a miRNA scaffold to enable more durable and efficient knockdown; for example, AAV5-miHTT targets exon 1, lowers mutant HTT in HD minipigs, improves motor phenotypes, and has advanced to early clinical evaluation [115]. Intrathecal administration is generally well tolerated, yet long-term efficacy and potential neuroinflammation require continued surveillance [112]. Moreover, endogenous noncoding RNAs, such as miR-196a, are dysregulated in HD and directly target HTT, suggesting a complementary strategy to restore network homeostasis using miRNA mimics or modulators [116,117].

### 3.4. ncRNA Dysregulation in ALS and Frontotemporal Dementia (FTD): Impacts on Protein Aggregation, miRNA Biogenesis, and RNA Homeostasis

In ALS and FTD, dysregulated ncRNAs drive protein aggregation and perturb RNA homeostasis. TDP-43 is a key regulator. Physiologically, it partners with the Drosha and Dicer complexes to orchestrate miRNA biogenesis [71]. When mislocalized to the cytoplasm, TDP-43 compromises miRNA processing, causing global miRNA downregulation and activation of pro-apoptotic pathways, thereby heightening neuronal vulnerability [118]. For example, miR-9 downregulation increases neurofilament light-chain expression and destabilizes axons [119]. Peripherally, skeletal-muscle miR-206 is upregulated as a compensatory response and targets HDAC4, transiently promoting neuromuscular-junction reinnervation; however, sustained elevation is insufficient to prevent motor neuron loss [120]. Together with miR-143 and miR-374b, miR-206 contributes to a serum biomarker panel with potential for early diagnosis [121]. At the genetic level, C9orf72 hexanucleotide repeat expansions generate toxic RNA foci and trigger repeat-associated non-ATG (RAN) translation, sequestering RNA-binding proteins and disrupting pre-mRNA splicing and RNA metabolism [122,123,124]. Antisense transcripts can form double-stranded RNA and activate innate immune signaling [125]. In FTD, miR-132 dysregulation is associated with tau pathology and cognitive impairment [126]. Antisense oligonucleotides targeting the repeat sequences reduce RNA foci and dipeptide-repeat products in model systems, highlighting ncRNAs as a shared pathological nexus and as candidate diagnostic and therapeutic targets across the ALS/FTD spectrum [127,128].

## 4. ncRNAs as Biomarkers

ncRNAs are recognized as central regulators in the pathogenesis of neurodegenerative diseases, modulating gene expression, epigenetic modifications, and cell-signaling pathways despite not encoding proteins. Their stability in biofluids renders them promising noninvasive biomarkers for early diagnosis, disease-progression monitoring, and therapeutic target discovery. However, establishing reliable ncRNA biomarkers still demands rigorous sample handling, sensitive assays, and robust statistical validation to minimize variability and ensure clinical translatability.

### 4.1. Sample Types and Preprocessing: Biofluid Sources, Handling Protocols, and Quality Control for Reliable ncRNA Detection

Across diseases, the reliability of ncRNA biomarkers is chiefly governed by the sample type and pre-analytical variables [129]. Plasma or serum is amenable to large-scale collection; however, a standardized, RNase-free workflow is essential: collect blood in K2-EDTA tubes; perform two-step centrifugation at 4 °C within 2 h of phlebotomy (1600× *g* for 10 min followed by 16,000× *g* for 10 min); aliquot into low-binding tubes and store at −80 °C [129]. Assess hemolysis by A414 or the miR-451a/miR-23a ratio and exclude outlier specimens [130]. CSF more directly reflects the central nervous system milieu [131]. Obtain CSF via sterile lumbar puncture, promptly centrifuge to remove cells, archive within 1 h, and record xanthochromia and cell counts as quality-control (QC) metrics [131]. To enhance brain-derived signals, neuron-derived extracellular vesicles (NDEVs) can be enriched [132]. Consistent with MISEV2023, physical methods such as size-exclusion chromatography or density-gradient separation are preferred, with multi-parametric characterization of particle size, protein markers, and nucleic-acid cargo; avoid sole reliance on L1CAM-based immunocapture, which may target soluble rather than vesicular material [133]. Across diseases, evidence indicates that in AD plasma levels of the lncRNAs BACE1-AS and NEAT1 are elevated and exhibit moderate diagnostic performance [134]; BACE1-AS stabilizes BACE1 mRNA, thereby increasing β-secretase activity [134,135]. In PD, serum miR-146a and miR-221 implicate inflammatory and neuronal-survival pathways [136]. In CSF, miR-132 and the lncRNA MALAT1 are associated with synaptic plasticity and tau phosphorylation [137]. Progressive supranuclear palsy (PSP) exhibits dysregulation of small non-coding RNAs accompanied by imbalances in tau isoforms [137]. In FTD, NDEVs are enriched for miR-124 and miR-146a, suggesting glial inflammation and aberrant APP processing [138]. Overall, extracellular-vesicle-borne ncRNAs amplify neurotoxicity and promote intercellular propagation through ceRNA networks, in which lncRNAs act as miRNA “sponges” to relieve repression of *APP*, *MAPT*, and related transcripts [138]. Accordingly, traceable and reproducible standard operating procedures with rigorous QC are indispensable for cross-center validation and clinical translation [132].

### 4.2. Detection Platforms: Comparative Analysis of qPCR, ddPCR, and NGS for ncRNA Quantification in Neurodegenerative Diseases

Building on optimized sample preparation, the selection of detection platforms for ncRNAs in NDDs must balance sensitivity for detecting low-abundance transcripts with scalability for high-throughput screening, while rigorous standardization—particularly under the updated MISEV2023 guidelines—mitigates inter-study variability and enhances reproducibility [132]. Traditional quantitative PCR (qPCR) serves as a cornerstone for the targeted quantification of known ncRNAs, such as plasma BACE1-AS in AD, providing cost-effective, high-sensitivity relative measurements normalized to stable references like GAPDH using the ΔΔCt method [139]; however, its limitations in absolute quantification and susceptibility to amplification biases have been addressed by droplet digital PCR (ddPCR), which partitions samples into thousands of droplets to enable precise, absolute quantification of low-abundance targets in EV-enriched samples, exhibiting superior performance in CSF lncRNA profiling for ALS since 2015 with reduced batch effects via spike-in controls [140]. In contrast, next-generation sequencing (NGS) excels in the unbiased discovery of novel ncRNA signatures, as exemplified by plasma sncRNA panels that predict AD with an AUC of 0.849, leveraging adapter-ligated libraries and bioinformatics tools such as DESeq2 to manage batch effects, albeit at higher costs and computational demands relative to PCR-based methods [141]; recent advancements have integrated NGS with ddPCR for hybrid validation, thereby enhancing accuracy in exosomal RNA analysis for AD [142]. MISEV2023 introduces key upgrades over previous versions, including new sections on EV release and uptake mechanisms as well as in vivo studies, mandating advanced isolation techniques such as differential ultracentrifugation or size-exclusion chromatography, coupled with multimodal characterization (e.g., TEM and Western blotting for CD63/TSG101), to prevent contamination from non-EV RNAs and ensure robust NDEV ncRNA data for NDD biomarkers. Critical evaluation of current methods reveals significant challenges: (1) exosome isolation protocols vary substantially across studies—differential ultracentrifugation (100,000× *g*, 70 min) yields different EV subpopulations than size-exclusion chromatography or immunoaffinity capture, leading to inconsistent ncRNA profiles from identical samples [143]; (2) the lack of universally accepted EV purity criteria means that “exosomal” miRNA signatures may be confounded by co-isolated protein-bound or lipoprotein-associated miRNAs; and (3) pre-analytical variables including blood collection tubes, processing delays, and freeze–thaw cycles disproportionately affect specific miRNA species, contributing to the poor reproducibility of circulating miRNA biomarkers across independent cohorts [144,145,146]. In summary, these platforms, when appropriately standardized, offer a comprehensive toolkit for ncRNA detection, thereby facilitating reliable data that can be robustly modeled and validated in subsequent analytical steps [132,147,148].

### 4.3. Machine Learning Approaches: Integration of ncRNA Data with Multimodal Models for Predictive Diagnostics and Mechanistic Insights

Following the generation of high-quality ncRNA data using standardized detection platforms, robust statistical modeling integrates multi-cohort datasets and multimodal inputs to achieve predictive accuracy, with an increasing emphasis on interpretable frameworks to elucidate complex interactions [149]. Conventional approaches, such as 70/30 data splits and 5-fold cross-validation, are employed to mitigate overfitting. However, critical methodological concerns persist: (1) most published ncRNA biomarker panels derive from discovery cohorts of *n* < 100, with feature-to-sample ratios exceeding 10:1 that virtually guarantee overfitting despite cross-validation; (2) fewer than 20% of studies include true external validation from independent centers, and those that do typically show substantial AUC degradation (0.85→0.65); (3) heterogeneous normalization strategies (global mean vs. reference genes vs. spike-ins) preclude cross-study comparison and may produce artifactual results. These limitations underscore the need for pre-registered analyses, larger multi-center cohorts, and standardized preprocessing protocols before ncRNA biomarkers can achieve clinical utility. As illustrated by elastic net regression, which identifies sncRNA signatures for MCI-to-AD conversion in 192 patients via feature penalization [141]; these models are calibrated using AUC, sensitivity/specificity, and net reclassification index (NRI) metrics, with Hosmer-Lemeshow tests ensuring model fit, yielding aggregated AUCs of 0.86 in plasma lncRNA meta-analyses for AD [150]. Advancing to multi-marker and multimodal integration through logistic regression combines ncRNAs with imaging (MRI) and clinical scores, as demonstrated by a 6-miRNA + ATN model achieving a C-statistic of 0.875 in AD, offering superior prognostic power over unimodal methods by capturing synergistic interactions [141]; however, traditional statistical approaches often lack the depth required to unravel intricate ceRNA networks [151]. Explainable artificial intelligence (XAI) represents a significant advancement, utilizing SHAP values and random forests to elucidate feature importance in ceRNA triads (e.g., BACE1-AS/miR-124/TP53) that drive AD/PD pathology [152,153], thereby providing transparency and mechanistic insights lacking in black-box deep learning models [154]; recent applications in multimodal frameworks include predicting AD cognitive status and differentiating AD from PD using interpretable neural networks [155,156], thus bridging statistical rigor with biological interpretability [149]. Overall, these modeling strategies not only enhance predictive capabilities but also foster a deeper mechanistic understanding, ultimately advancing ncRNA-based diagnostics toward clinical utility in NDDs [157]. However, significant limitations persist: the majority of ML-derived ncRNA signatures have not been validated in external cohorts; overfitting risks are substantial given typical sample sizes (<200 subjects) relative to feature dimensionality (thousands of ncRNAs); and “black box” deep learning models lack the interpretability required for clinical adoption and regulatory approval. The field must transition from discovery-focused exploratory analyses to hypothesis-driven validation studies with pre-specified endpoints and independent test cohorts. Key circulating non-coding RNAs that have been proposed as diagnostic or prognostic biomarkers for Alzheimer’s disease and Parkinson’s disease are listed in Table 2.

To address these limitations and accelerate clinical translation, the field urgently requires: (1) prospective, multi-center validation studies with pre-registered analysis plans; (2) standardized sample processing and normalization protocols aligned with MISEV2023 and MIQE guidelines; (3) open sharing of raw data and analysis code to enable independent replication; (4) application of explainable AI (XAI) methods that provide biological interpretability beyond statistical prediction; and (5) integration of ncRNA signatures with established protein biomarkers and clinical scores rather than standalone diagnostic use [161].

## 5. From Mechanism to Therapy: RNA Interventions and Delivery

### 5.1. Therapeutic Modalities: The RNA Intervention Spectrum and Mechanisms from ASO/siRNA to CRISPRi/a

Rapid advances in understanding non-coding RNA dysregulation in neurodegenerative diseases have yielded a diverse toolkit of RNA-targeted modalities that operate at multiple levels of RNA metabolism [77,162,163]. Antisense oligonucleotides (ASOs) with phosphorothioate backbones and 2′-O-modified sugars represent the most clinically advanced platform, enabling sequence-specific binding to pre-mRNA or mRNA to modulate splicing, induce RNase H–mediated degradation, or sterically block translation [162,163]. “Gapmer” ASOs, which incorporate a central DNA core flanked by modified ribose wings, are particularly efficient at recruiting RNase H1 and are therefore well suited for targeting toxic gain-of-function transcripts, such as mutant SOD1, MAPT, or HTT, where partial knockdown is desirable [77,163,164,165]. In parallel, double-stranded siRNAs and shRNA/miRNA cassettes engage the endogenous RNA-induced silencing complex (RISC) to cleave target RNAs at Argonaute-defined sites, a mechanism increasingly exploited for CNS indications that were once considered inaccessible to RNA interference (RNAi) [166,167]. Synthetic miRNA mimics extend this approach to multitarget regulation, whereas chemically stabilized anti-miRs (antagomirs) sequester disease-promoting miRNAs, providing an attractive strategy in settings where non-coding RNAs themselves constitute pathogenic nodes [168]. Long non-coding RNAs (lncRNAs), which scaffold chromatin modifiers, modulate RNA processing, and act as competing endogenous RNAs, are now recognized as key regulators in AD, PD, ALS, and HD [169]. Beyond ASO- or siRNA-mediated knockdown, CRISPR interference/activation (CRISPRi/a) platforms tether catalytically dead Cas9 or RNA-targeting Cas13 to repressive or activating domains, thereby enabling sustained transcriptional silencing or activation of lncRNA loci and selective targeting of nuclear or cytoplasmic lncRNA transcripts without introducing DNA breaks [170,171]. Collectively, these modalities provide a continuum ranging from transient post-transcriptional silencing (siRNA, miRNA mimics/antagomirs), through semi-durable RNase H–mediated degradation (gapmer ASOs), to long-lasting transcriptional modulation and RNA editing achieved via viral vectors and CRISPR-based systems, which can in principle be tailored to the allelic architecture and cell type–specificity of individual neurodegenerative disorders [77,163,166,171].

### 5.2. Clinical and Preclinical Evidence: Translational Progress Across ALS, Tauopathy, and HD

Multiple mechanistically informed programs demonstrate the translational potential of RNA-based therapies in neurodegenerative disease [77,172,173]. The SOD1-directed antisense oligonucleotide (ASO) tofersen, administered intrathecally in SOD1-associated ALS (SOD1-ALS), reduced CSF SOD1 by ~30–40% in the highest-dose cohorts of a phase 1/2 trial and lowered plasma neurofilament light chain (NfL) [173]. In the subsequent VALOR phase 3 study, tofersen consistently suppressed CSF SOD1 and plasma NfL and, when initiated earlier, was associated with a slower decline in ALS Functional Rating Scale–Revised (ALSFRS-R) scores, supporting a presymptomatic, biomarker-guided paradigm despite not meeting the primary clinical endpoint in the initial 28-week analysis [77,174]. Its accelerated approval by the FDA in April 2023 relied on plasma NfL reduction as a reasonably likely surrogate endpoint, representing the first regulatory use of a fluid biomarker of neurodegeneration to support an RNA therapeutic [174] and underscoring the linkage between target engagement and axonal integrity [172,175].

A parallel narrative is emerging for tau pathology: the MAPT-targeting ASO BIIB080 (MAPTRx) achieved approximately 50% reductions in CSF total tau and p-tau181 in a phase 1b trial in patients with mild AD (Mummery et al., 2023 [164], presented at CTAD). These reductions persisted for at least 12 weeks after the last dose. Tau-PET exploratory analyses showed numerical reductions in signal in drug-treated groups, though the study was not powered for clinical outcomes [164]. These findings provide an in-human bridge between tau lowering, fluid biomarkers, and imaging readouts, supporting tau as a tractable RNA-level target [164]

In Huntington’s disease, a phase 1/2a study of the HTT-lowering ASO tominersen showed dose-dependent reductions in CSF mutant huntingtin, but phase 3 futility highlighted risks of oversuppression and suboptimal dosing [112,176]. Critical analysis of the tominersen failure reveals several mechanistic lessons: (1) non-selective targeting of both mutant and wild-type HTT may have depleted neuroprotective wild-type huntingtin functions, suggesting that allele-selective approaches should be prioritized; (2) the chosen dosing interval (every 8 weeks) may have achieved excessive cumulative CNS exposure, as CSF mHTT suppression exceeded 40%, potentially below the threshold for normal cellular function; (3) post hoc analyses suggest that younger patients with less disease burden showed worse outcomes, challenging the assumption that earlier intervention is always beneficial for gene-silencing therapies; and (4) the lack of validated pharmacodynamic biomarkers beyond CSF mHTT prevented early detection of adverse neural effects [177]. These lessons underscore that target engagement alone is insufficient—safety margins and biological context must guide dosing strategies for RNA therapeutics. This experience shifted development toward more sustained, regionally restricted strategies, such as AAV5-miHTT (AMT-130), which delivers a microRNA targeting HTT to striatal and cortical neurons. In preclinical models, AAV5-miHTT produced widespread distribution in striatum and cortex, durable HTT mRNA and protein lowering (60–80% reduction sustained for at least 12 months), and functional rescue with limited off-target effects [178,179]. Building on these data, first-in-human phase 1/2 trials are evaluating single-dose neurosurgical administration of AMT-130 to assess safety, target engagement, and potential disease-modifying effects in patients with Huntington’s disease [179,180,181]. Collectively, these ALS, tauopathy, and Huntington’s disease programs show how mechanistically informed RNA interventions can be paired with fluid and imaging biomarkers to de-risk development and move toward disease modification in conditions long regarded as untreatable. Current RNA-based therapeutic strategies that target non-coding RNAs or their downstream pathways in neurodegenerative diseases are outlined in Table 3.

### 5.3. Brain Delivery Strategies: Comparing Intrathecal/Intraventricular Routes, Receptor-Mediated BBB Shuttles, and Exosome Carriers

The success of nucleic acid-based interventions is constrained by the ability to deliver cargos to widely distributed neuronal and glial populations in the human brain [182]. Currently, intrathecal (IT) or intracerebroventricular (ICV) administration is the workhorse route for ASOs and some RNA-expressing viral vectors, exploiting cerebrospinal fluid (CSF) flow to bathe the neuraxis but generating rostral–caudal and gray–white gradients that preferentially expose the spinal cord and periventricular regions [183]. Repeated IT dosing, as with tofersen or BIIB080, permits titration and discontinuation but requires serial lumbar punctures and carries cumulative risk of aseptic meningitis, radiculitis and CSF pressure perturbations [77,164,183]. In contrast, parenchymal neurosurgical delivery, exemplified by AMT-130, can achieve high local concentrations and durable expression in deep nuclei, but is anatomically restricted, irreversible, and dependent on precise stereotactic planning and real-time imaging [178]. To widen the therapeutic window and reduce procedural burden, efforts are focused on receptor-mediated transcytosis across the blood–brain barrier (BBB), typically by fusing therapeutic cargos or nucleic acid–binding scaffolds to antibodies against endothelial receptors such as transferrin receptor 1 (TfR1) or CD98hc [182,184]. Recent work shows that bispecific antibody shuttles targeting CD98hc enable more efficient and longer-lived brain uptake of immunoglobulins than classical TfR1 shuttles, with distinct partitioning between neuronal and vascular compartments and a more favorable safety window [185,186]. Systematic analyses further demonstrate that receptor affinity, valency and epitope strongly influence the balance between endothelial uptake and transcytosis, indicating that optimized “brain shuttle” architectures will be required to deliver oligonucleotides, lipid nanoparticles or exosome-like vesicles into the parenchyma at clinically meaningful doses [184]. In parallel, exosomes and engineered extracellular vehicles (EVs) are emerging as biological carriers for RNA drugs [187,188,189]. Neuronal- or dendritic cell-derived exosomes engineered to display the rabies virus glycoprotein (RVG) peptide can encapsulate siRNAs or miRNA mimics and, following systemic administration, deliver their cargo to neurons, microglia and oligodendrocytes across the BBB, modulating targets such as BACE1 or α-synuclein in preclinical models [187,190]. Compared with synthetic nanoparticles, exosomes show favorable biodistribution, lower immunogenicity and enhanced endosomal escape; however, challenges in manufacturing, loading efficiency and control of tropism remain, and will be critical to resolve for clinical translation in neurodegenerative disease [188,189].

### 5.4. Safety and Quality—Innate Immune Activation, Organ Burden, CMC Consistency, and Biomarker Monitoring

As sophisticated delivery platforms push RNA therapeutics deeper into the CNS, safety and quality are central [191,192,193]. Oligonucleotides can activate innate immune pathways through endosomal Toll-like receptors, cytosolic nucleic-acid sensors and complement, producing inflammatory and hematologic toxicities, with risks shaped by backbone chemistry and dosing regimen [192,193]. Liver and kidney are key organs for clearance, and even CSF-delivered antisense oligonucleotides ultimately reach peripheral tissues, requiring monitoring of liver enzymes, coagulation, renal function and complement activity [191,194,195]. Hybridization-dependent off-target effects from partial complementarity to unintended transcripts can cause unanticipated toxicities alongside on-target knockdown, so development pipelines now incorporate in silico design, transcriptome-wide off-target screening and tiered non-clinical toxicology packages [192]. For viral RNA-expressing vectors, stringent control of vector genome integrity, replication-competent virus, capsid impurities and host–cell protein carryover is required to maintain batch-to-batch consistency in potency and immunogenicity [191,192].

Clinically, rebound or complex biomarker changes after treatment interruption—such as the unfavorable clinical outcomes seen in some dosing regimens in tominersen trials—have underscored that long-term pharmacodynamics and dosing intervals must be aligned with target protein turnover and the resilience of the affected neural circuits [112,176,191]. Within this framework, quantitative biomarkers are central to monitoring efficacy and safety, including neurofilament light and phosphorylated neurofilament heavy chain as markers of axonal injury, disease-specific CSF biomarkers (SOD1, tau, mutant HTT), imaging readouts and digital measures of motor and cognitive function [77,196]. The tofersen experience illustrates how sustained lowering of a neuronal injury biomarker can serve as a mechanistically grounded surrogate endpoint when linked to robust target engagement [77], while ongoing tau- and HTT-lowering programs will test whether similar biomarker frameworks can extend across proteinopathies [112,164,176]. Ultimately, the path from mechanism to therapy in non-coding RNA biology is bidirectional, as mechanistic insights guide RNA drug design and placement, and clinical results feed back into questions about RNA metabolism and cell-type vulnerability in the aging brain [164,192].

## 6. Key Challenges and Future Prospects

The foremost challenge in ncRNA research for neurodegenerative diseases stems from substantial heterogeneity in cell types, brain regions, and disease stages. Even among patients with the same clinical diagnosis, neurons and glial cells undergo distinct transcriptional reprogramming, yielding ncRNA expression profiles that are highly cell-specific and temporally dynamic [90,197]. Bulk tissue or biofluid analyses inevitably average signals from vulnerable and resilient cell populations, so the same ncRNA may appear as a “pathogenic driver” in one cohort but a “terminal stress byproduct” in another [90]. Such context-dependent findings complicate molecular subtyping and hamper target prioritization in these disorders. An additional pivotal challenge is the multifaceted interaction network linking ncRNAs, RBPs, and epitranscriptomic modifications. Chemical modifications like N^6-methyladenosine (m^6A) and adenosine-to-inosine editing can modulate miRNA biogenesis, alter lncRNA structure, and affect circRNA stability, while also redefining RBP binding affinities and their propensity for liquid–liquid phase separation [3,198]. Disruption of this finely tuned network (for example, by aberrant m^6A levels or RBP mutations) can trigger downstream pathologies such as protein aggregation, synaptic dysfunction, or shifts in cell fate [3]. Notably, m^6A dysregulation has been linked to pathological condensates in neurodegenerative disease [198]. However, integrative quantitative models that unify these regulatory layers are still lacking. To address this gap, we propose several testable research directions: (1) systematic single-cell multiome profiling (RNA-seq + ATAC-seq + m6A-seq) across brain regions and disease stages to construct cell type-specific ncRNA regulatory networks; (2) CRISPR-based functional screens in human iPSC-derived neurons and microglia to establish causal ncRNA–phenotype relationships; (3) development of computational frameworks that integrate ncRNA expression, RBP binding (from eCLIP data), and epitranscriptomic modifications to predict functional ncRNA–target interactions; and (4) establishment of ncRNA “perturbation atlases” cataloging the transcriptome-wide effects of individual ncRNA knockdown/overexpression to distinguish primary from secondary regulatory relationships.

Methodologically, systematic biases in sample handling and data normalization are major impediments to reproducing ncRNA biomarkers. Pre-analytical variables—including the extent of hemolysis, centrifugation protocols, freeze–thaw cycles, and choice of anticoagulant—significantly influence small RNA yields and exosomal content in plasma or cerebrospinal fluid [144]. Moreover, inconsistent use of exogenous spike-in controls, endogenous reference genes (e.g., U6, small nuclear RNAs, or presumed “stable” miRNAs), and normalization methods (global mean, quantile normalization, etc.) often leads to divergent expression trends for the same circulating miRNA across studies [199,200]. For instance, different normalization strategies can produce conflicting results from identical qPCR panels, emphasizing how critical the choice of method is to reliable interpretation [199]. In addition, many machine learning-derived multi-miRNA panels from small discovery cohorts suffer from overfitting and lack external validation, which—combined with opaque “black box” algorithms—undermines their generalizability and regulatory acceptance. Indeed, inconsistencies in the literature and standard assay protocols have limited the clinical application of miRNA biomarkers to date [200]. Consequently, establishing standardized preprocessing protocols, appropriate reference controls with quality metrics [144], uniform reporting guidelines, independent validation cohorts, and explainable AI models is essential to transition ncRNA biomarkers from exploratory research to robust clinical evidence [200].

Regarding translational applications, crossing the BBB while ensuring long-term safety is the primary obstacle for ncRNA-based therapeutics. Intrathecal antisense oligonucleotides—exemplified by nusinersen for spinal muscular atrophy and tofersen for SOD1-mutant ALS—have provided proof-of-concept that modulating RNA in the central nervous system can slow disease progression [201,202]. However, these drugs require repeated lumbar punctures or implanted infusion devices, and they carry significant risks including dorsal root ganglion toxicity, thrombocytopenia, and renal injury [201,202]. Systemic delivery approaches (intravenous or subcutaneous) using chemically modified oligonucleotides packaged in lipid nanoparticles or conjugated to receptor-targeting ligands offer the promise of broader brain biodistribution with less invasiveness [203]. Still, rigorous evaluation in aged, comorbid patient populations is needed to monitor immune stimulation, off-target tissue accumulation, and potential tumorigenicity over prolonged use. Specific solutions emerging from recent advances include: (1) next-generation ASO chemistries (e.g., constrained ethyl modifications) that improve CNS distribution after systemic administration; (2) brain-penetrant antibody–oligonucleotide conjugates leveraging transferrin receptor or CD98hc transcytosis, with optimized affinity/valency for enhanced parenchymal delivery; (3) endogenously produced EV-based delivery systems engineered from patient-derived cells to minimize immunogenicity; and (4) focused ultrasound-mediated BBB opening combined with microbubble-loaded ncRNA therapeutics for spatially targeted CNS delivery—an approach currently in early clinical testing for brain tumors and potentially applicable to neurodegenerative diseases.

Prospectively, transcriptome-based “endotypes” defined by ncRNA profiles may provide new axes to stratify the clinical heterogeneity of neurodegenerative diseases. Studies across multiple cohorts suggest that patients can be grouped by co-expression networks of miRNAs, lncRNAs, and circRNAs into biologically coherent subtypes—for example, an inflammation-predominant subtype, a synaptic dysfunction subtype, or a mitochondrial/metabolic stress subtype [204]. These ncRNA-driven clusters can be combined with other omics signatures (e.g., genetic risk variants, proteomic and metabolomic patterns) to refine patient stratification, improve prognostic models, and predict therapeutic responses. Identifying such ncRNA modules and their key hub regulators in each subgroup could guide personalized therapeutic strategies—for instance, selective anti-inflammatory ncRNA targets in the neuroinflammatory subgroup versus synapse-protective targets in the synaptic-disruption subgroup.

On the technological front, merging single-cell sequencing with long-read platforms is enabling unprecedented resolution of ncRNA biology at the isoform level within individual cells. Using single-cell long-read RNA sequencing, researchers have begun to map full-length transcript isoforms (including alternative splicing and polyadenylation variants) in distinct brain cell types and regions [205]. These approaches reveal extensive cell-type- and region-specific isoform regulation—affecting protein architecture and disease-associated variants—that was previously hidden by short-read methods [205]. Coupling such transcriptomic precision with spatial transcriptomics now permits the reconstruction of ncRNA–RBP–modification interactomes in situ, across intact neural circuits. Spatially resolved transcriptomics retains information on the local tissue architecture and molecular microenvironment, allowing dysregulated ncRNA networks to be mapped in the context of pathogenic lesions (for example, around protein aggregates or neuroinflammatory foci) [205]. By integrating these cutting-edge tools, we can pinpoint actionable molecular targets in specific cell subpopulations or circuit locations. This paves the way for multimodal interventions—for instance, combining an ncRNA therapeutic (such as a targeted antimiR or lncRNA mimic) with a small-molecule inhibitor, antibody, or cell therapy—to simultaneously correct converging pathogenic pathways identified by the interactome maps.

With the maturation of multi-omics data integration, explainable artificial intelligence, and supportive regulatory frameworks for nucleic-acid drugs, ncRNAs are poised to evolve from simple correlational biomarkers to dual-purpose agents that serve as both etiological targets and precision-guided diagnostic tools. In the coming years, we expect ncRNAs to be leveraged not only to detect and monitor neurodegenerative diseases, but also to subtype patients mechanistically and to tailor therapies accordingly. The convergence of single-cell multi-omics and spatial analyses is enriching our understanding of disease mechanisms, while advanced computational models help translate these insights into clinically actionable knowledge. These innovations, together with growing experience in the safe delivery of RNA therapeutics, herald a new era in which ncRNA-based interventions could be deployed to halt or even prevent neurodegenerative disorders—fulfilling their potential as “bifunctional” entities that inform both the biology and treatment of these devastating diseases [205].

## 7. Conclusions

Non-coding RNAs have moved from the periphery to the center of neurodegeneration research, reshaping our understanding of how gene-regulatory networks fail in Alzheimer’s disease, Parkinson’s disease, Huntington’s disease, amyotrophic lateral sclerosis, and related disorders. Across these conditions, miRNAs, lncRNAs, circRNAs, and piRNAs converge on synaptic plasticity, proteostasis, mitochondrial fitness, neuroinflammation, and genome stability, thereby linking diverse genetic and environmental insults to shared pathogenic pathways. At the same time, their remarkable stability in biofluids and extracellular vesicles has positioned ncRNAs as promising circulating and tissue-based biomarkers for early diagnosis, disease monitoring, and patient stratification.

In summary, this review highlights five key findings: (1) ncRNAs—including miRNAs, lncRNAs, circRNAs, and piRNAs—regulate core pathogenic processes across major neurodegenerative diseases through mechanistically distinct but convergent pathways affecting synaptic function, proteostasis, mitochondrial homeostasis, and neuroinflammation; (2) circulating ncRNA biomarkers show diagnostic promise, though clinical translation requires standardized methodologies and independent validation; (3) RNA-targeting therapeutics have achieved clinical proof-of-concept with FDA approval of tofersen for SOD1-ALS, while failures such as tominersen provide critical lessons for future development; (4) integration of ncRNA biology with RBP function and epitranscriptomic regulation represents an essential but underexplored frontier; and (5) emerging technologies including single-cell and spatial transcriptomics are poised to resolve cell type-specific ncRNA dysregulation and guide precision therapeutic development.

Yet key challenges remain. Cell-type and stage-specific heterogeneity of ncRNA expression, incomplete integration of RBPs and epitranscriptomic modifications, and variability in sample handling and data analysis continue to limit reproducibility and clinical translation. Overcoming blood–brain barrier constraints and ensuring long-term safety of RNA therapeutics in aged, comorbid populations will be equally critical. Looking ahead, combining single-cell and spatial transcriptomics with explainable artificial intelligence is expected to reveal mechanistically defined ncRNA endotypes and actionable network hubs, opening the door to rational patient stratification and combination therapies.

Taken together, ncRNAs are poised to function as “bifunctional” entities in neurodegenerative diseases—serving simultaneously as high-resolution molecular readouts and as levers for precise intervention. Systematic efforts to harmonize methodology, deepen mechanistic insight, and embed ncRNA metrics in clinical trial design will be essential to translate this promise into durable neuroprotection and, ultimately, disease prevention.

## Figures and Tables

**Table 1:** Representative non-coding RNAs with mechanistic roles in Alzheimer’s disease and Parkinson’s disease.

Disease	ncRNA (Type)	Primary Target/Pathway	Change in Disease Context	Key Functional Effect	Model/Sample	Evidence Strength	Key Knowledge Gaps	Refs.
Alzheimer’s disease	miR-132 (microRNA)	Regulates networks controlling adult hippocampal neurogenesis and synaptic plasticity	Consistently downregulated in hippocampus of AD patients and mouse models	miR-132 replacement restores adult hippocampal neurogenesis and rescues memory deficits in AD mouse models	Multiple AD mouse models, human neural stem cells, post-mortem human hippocampus	Strong	Optimal delivery method for clinical translation undefined; long-term safety of miR-132 restoration unknown	[82]
Alzheimer’s disease	BACE1-AS (lncRNA, antisense to BACE1)	BACE1 mRNA/BACE1 protein and amyloidogenic processing	BACE1-AS expression is altered in STZ-induced AD rats and further increased after memantine treatment	Changes in BACE1-AS in brain and blood parallel alterations in BACE1 protein, suggesting roles in AD pathogenesis and treatment monitoring	STZ-induced AD rat model, brain and blood tissues	Moderate	Causality vs. correlation unclear; human validation limited to postmortem tissue	[103]
Alzheimer’s disease	miR-29a/b/c (microRNA)	BACE1 3′UTR/β-secretase activity	Downregulated in sporadic AD brain tissue	Reduced miR-29 leads to elevated BACE1, increased Aβ production, and plaque formation	Human postmortem brain, cell lines, AD mouse models	Strong	Specificity of miR-29 targeting; potential off-target effects of miR-29 mimics	[78,79]
Alzheimer’s disease	miR-34c (microRNA)	SIRT1, SYT1/survival and synaptic pathways	Upregulated in AD hippocampus	Promotes apoptosis, mitochondrial stress, and memory impairment	AD mouse models, human postmortem tissue	Moderate	Direction of causality unclear; functional redundancy with miR-34a/b	[80,81]
Alzheimer’s disease	NEAT1 (lncRNA)	FZD3/GSK3β/p-tau axis; paraspeckle formation	Upregulated in AD models	Modulates microtubule stability and tau phosphorylation; context-dependent neuroprotective or pathogenic roles	SH-SY5Y cells, APP/PS1 mice	Moderate	Conflicting reports on protective vs. deleterious effects; cell type-specific functions undefined	[47,48]
Parkinson’s disease	miR-7 (microRNA)	SNCA (α-synuclein) and autophagy machinery	miR-7 downregulation contributes to α-synuclein accumulation	miR-7 overexpression promotes autophagic clearance of α-synuclein monomers and aggregates, protecting against α-synuclein-induced toxicity	Differentiated human ReNcell VM cells overexpressing α-synuclein	Moderate	No clinical trial data; optimal delivery route to substantia nigra undefined	[104]
Parkinson’s disease	miR-7 (microRNA)	NLRP3/caspase-1 inflammasome and neuroinflammation	α-Synuclein overexpression reduces miR-7 and activates NLRP3 inflammasome	Stereotactic delivery of miR-7 mimics inhibits NLRP3/caspase-1 activation and improves subventricular zone neurogenesis	A53T α-synuclein transgenic mice; adult neural stem cells; intracerebroventricular miR-7 mimics	Moderate	Intracerebroventricular delivery not practical for chronic therapy; systemic delivery challenges	[105]
Parkinson’s disease	miR-34b/c (microRNA)	DJ-1, Parkin/mitophagy and mitochondrial quality control	Downregulated in PD substantia nigra	Compromises mitochondrial quality control and produces energetic deficits	Human postmortem substantia nigra, cell lines	Preliminary	Single postmortem study; functional validation limited	[97]
Parkinson’s disease	circSNCA (circRNA)	miR-7 sponging/SNCA expression	Elevated in PD models	circSNCA elevation suppresses miR-7, increases SNCA, promotes autophagy suppression and redox stress	Cell models, pramipexole treatment studies	Preliminary	Limited to cell culture; in vivo validation needed	[95,96]

Evidence Strength Criteria: Strong: Validated in multiple independent human cohorts AND mechanistic studies in multiple model systems. Moderate: Animal model validation with limited human correlative data OR multiple cell culture studies. Preliminary: Single studies, cell culture only, or conflicting results across studies.

**Table 2:** Circulating non-coding RNA biomarkers in Alzheimer’s disease and Parkinson’s disease.

Disease	ncRNA(s)	Sample Type	Direction of Change	Diagnostic Performance/Clinical Association	Study Population/Notes	Validation Status	Methodological Quality	Key Limitations	Refs.
Alzheimer’s disease	BACE1 (lncRNA)	Plasma	Increased in AD versus non-AD patients	Plasma BACE1 level showed high specificity (≈88%) for discriminating AD from non-AD subjects	Case–control study comparing four lncRNAs in plasma of AD and non-AD individuals; BACE1 showed the most robust separation	Discovery only	Moderate (QUADAS-2)	Single-center; no external validation; small sample size	[135]
Alzheimer’s disease	BACE1-AS (lncRNA)	Plasma and plasma-derived exosomes	Overall AD vs. control difference modest; but BACE1-AS levels differ between pre-AD and full AD subgroups	Plasma BACE1-AS discriminates pre-AD and controls, full AD and controls, and pre-AD and full AD with high specificity in ROC analyses	Cross-sectional cohort including AD, pre-AD and cognitively normal controls; BACE1-AS quantified in plasma and exosomes	Internal validation	Moderate (QUADAS-2)	Cross-sectional design; exosome isolation method variability	[134]
Alzheimer’s disease	Exosomal BACE1-AS (lncRNA)	Plasma exosomes	Elevated in AD compared with controls	Exosomal BACE1-AS alone yields AUC ≈ 0.76; combining exosomal BACE1-AS with right entorhinal cortex volume and thickness increases AUC and improves both sensitivity and specificity	AD patients and controls with plasma exosome profiling and 3D MRI of entorhinal cortex; combined molecular–imaging signature proposed as detection biomarker	Internal validation	Moderate (QUADAS-2)	Requires MRI integration; exosome isolation not standardized	[158]
Alzheimer’s disease	miR-29a, miR-125b panel (microRNA)	Serum/plasma exosomes	Altered in AD	Multi-analyte panels combining exosomal miRNAs improve diagnostic accuracy with reported sensitivities >80%	Multiple cohort studies	Discovery + partial validation	Low-Moderate (QUADAS-2)	Heterogeneous methods across studies; no consensus panel	[85,86,87]
Alzheimer’s disease	miR-135a (microRNA)	CSF, plasma	Altered in AD progression	Tracks disease progression in longitudinal studies	Longitudinal cohort studies	Internal validation	Moderate (QUADAS-2)	Limited sample sizes; normalization variability	[141]
Parkinson’s disease	miR-221 (microRNA)	Serum	Decreased in PD relative to healthy controls	Serum miR-221 shows ROC AUC ≈ 0.79 and correlates positively with UPDRS-III and UPDRS-V scores, suggesting association with motor severity	138 PD patients and 112 controls; qRT–PCR panel of 16 PD-related miRNAs measured in serum	Internal validation	Moderate (QUADAS-2)	Single-center; no external validation cohort	[136]
Parkinson’s disease	miR-195, miR-185, miR-15b, miR-221, miR-181a (microRNA panel)	Serum	miR-195 upregulated; miR-185, miR-15b, miR-221 and miR-181a downregulated in PD	Five-miRNA signature accurately distinguishes PD from healthy individuals and is proposed as a serum-based diagnostic panel	Discovery and validation cohorts of PD patients and controls; high-throughput qRT–PCR and ROC analysis	External validation	High (QUADAS-2)	Validation cohort from same geographic region; generalizability uncertain	[159]
Parkinson’s disease	miR-214, miR-221, miR-141 (microRNAs, combined with cytokines and antioxidants)	Serum	miR-214 decreased; miR-221 and miR-141 decreased together with altered cytokines and antioxidant markers	Combined panel of miRNAs, cytokines, α-synuclein and antioxidant markers improves discrimination between PD patients and controls compared with single markers	20 PD patients and 15 controls; integrated analysis of serum cytokines, α-synuclein, miRNAs and antioxidant enzymes	Discovery only	Low (QUADAS-2)	Very small sample size (*n* = 35); no validation	[160]
Parkinson’s disease	EV-miRNAs (miR-7, miR-153, miR-19b)	CSF, serum EVs	Altered levels correlating with PD severity	Panels integrating EV-miRNAs with circRNAs show promising early diagnostic performance	Longitudinal and cohort studies	Internal validation	Moderate (QUADAS-2)	EV isolation heterogeneity; study-specific panels	[99,100,101]

Validation Status Criteria:—Discovery only: Initial identification in a single cohort without independent testing—Internal validation: Cross-validation or hold-out testing within the same study population—External validation: Independent validation in a separate cohort from a different center/population. Methodological Quality (QUADAS-2 Assessment): High: Low risk of bias across all domains; consecutive/random patient selection; blinded interpretation. Moderate: Some concerns in 1–2 domains; adequate patient selection but potential spectrum bias. Low: High risk of bias in multiple domains; convenience sampling; unblinded interpretation.

**Table 3:** RNA-based therapeutic strategies targeting non-coding RNAs or their pathways in neurodegenerative diseases.

Disease/Indication	Therapeutic Strategy	Target RNA/Pathway	Delivery Approach	Key Preclinical or Clinical Outcome	Development Stage	Evidence Level	Key Limitations/Failures	Refs.
Alzheimer’s disease	miR-132 replacement therapy	miR-132 deficiency affecting adult hippocampal neurogenesis and memory circuits	Hippocampal delivery of miR-132 (e.g., viral vectors or mimics) in AD mouse models	Restoring miR-132 expression rescues adult hippocampal neurogenesis and ameliorates memory deficits in AD mouse models, supporting miR-132 as a therapeutic candidate	Preclinical (mouse models, human neural stem cells)	Preclinical (in vivo)	No human safety data; delivery to human hippocampus technically challenging; long-term expression stability unknown	[82]
Alzheimer’s disease	BACE1-AS targeting	BACE1-AS lncRNA/BACE1 stabilization	ASO or siRNA targeting BACE1-AS	Knockdown reduces BACE1 levels and Aβ production in cell models	Preclinical (cell culture)	Preclinical (in vitro)	Cell culture only; in vivo validation lacking; potential off-target effects on BACE1 regulation	[42,43]
Alzheimer’s disease	Anti-miR-34c therapy	miR-34c/SIRT1, synaptic targets	Antagomir delivery	Inhibition of miR-34c rescues memory deficits in AD mouse models	Preclinical (mouse models)	Preclinical (in vivo)	Specificity concerns (miR-34 family redundancy); no human data	[84]
Parkinson’s disease	miR-7 overexpression to reduce α-synuclein burden	SNCA (α-synuclein) and autophagy pathway	Lentiviral miR-7 overexpression in neuron-like cells (ReNcell VM) expressing α-synuclein	miR-7 reduces monomeric and aggregated α-synuclein and promotes autophagic clearance, highlighting miR-7 as a potential disease-modifying approach	Preclinical (human neuron-like cell model)	Preclinical (in vitro)	Cell model only; no in vivo PD model testing; delivery to substantia nigra undefined	[104]
Parkinson’s disease	miR-7 mimic administration to modulate neuroinflammation and neurogenesis	NLRP3/caspase-1 inflammasome and α-synuclein–induced neuroinflammation	Stereotactic intracerebroventricular injection of synthetic miR-7 mimics in A53T α-synuclein transgenic mice	miR-7 mimics suppress NLRP3/caspase-1 activation and improve subventricular zone neurogenesis, suggesting potential to restore regenerative capacity in PD	Preclinical (transgenic mouse model)	Preclinical (in vivo)	Invasive delivery (ICV injection); chronic dosing not evaluated; translation to human unclear	[105]
Parkinson’s disease	circSNCA modulation	circSNCA/miR-7 sponging	Pharmacological (pramipexole)	Pramipexole downregulates circSNCA, restores miR-7, and attenuates apoptosis	Preclinical (cell models)	Preclinical (in vitro)	Indirect mechanism; pramipexole’s circSNCA effects may be secondary to dopaminergic action	[95,96]
SOD1-mutant ALS	Antisense oligonucleotide tofersen	SOD1 mRNA (reducing mutant SOD1 protein)	Repeated intrathecal administration of ASO (100 mg)	Phase 3 trial shows tofersen lowers CSF SOD1 and plasma neurofilament light; early-start treatment in the open-label extension is associated with more favorable functional outcomes despite primary endpoint not being met	FDA Approved (April 2023)	Phase 3 + Approved	Primary clinical endpoint not met in initial analysis; requires repeated lumbar punctures; limited to SOD1 mutation carriers (~2% of ALS)	[77,173]
Tauopathy/mild AD	MAPT-targeting ASO (BIIB080/MAPTRx)	MAPT mRNA/tau protein	Intrathecal administration	~50% reduction in CSF total tau and p-tau181; reductions persist ≥12 weeks post-dosing; numerical tau-PET reductions	Phase 1b completed; Phase 2 ongoing	Phase 2	Not powered for clinical outcomes; long-term safety unknown; requires repeated IT dosing	[164]
Huntington’s disease	HTT-lowering ASO tominersen	HTT mRNA (both mutant and wild-type)	Intrathecal administration	Phase 1/2a showed dose-dependent CSF mHTT reduction; Phase 3 halted for futility with worse outcomes in some treatment groups	Phase 3 (terminated)	Phase 3 (Failed)	CRITICAL FAILURE: Non-allele-selective; possible over-suppression of wild-type HTT; younger patients showed worse outcomes; dosing interval may have been suboptimal	[112,176]
Huntington’s disease	AAV5-miHTT (AMT-130) gene therapy	HTT mRNA via artificial microRNA	Single neurosurgical intrastriatal injection	Preclinical: 60–80% HTT mRNA/protein reduction sustained ≥12 months in rodents and NHPs with functional rescue; Phase 1/2 ongoing	Phase 1/2 (ongoing)	Phase 1/2	Irreversible; requires neurosurgery; long-term safety of permanent HTT suppression unknown; phase 3 tominersen failure raises caution	[178,179]

Evidence Level Criteria: Preclinical (in vitro): Cell culture studies only. Preclinical (in vivo): Animal model studies with therapeutic intent. Phase 1/2: Early clinical trials assessing safety and preliminary efficacy. Phase 2: Clinical trials with efficacy signals. Phase 3: Pivotal efficacy trials. Phase 3 (Failed): Pivotal trials that did not meet primary endpoints. Approved: Regulatory approval obtained.

## Data Availability

No new data were created or analyzed in this study.

## Abbreviations

The following abbreviations are used in this manuscript:

3′UTR	3′ untranslated region
AAV	adeno-associated virus
AAV-miRNAs	adeno-associated virus vectors expressing artificial microRNAs
AD	Alzheimer’s disease
ADAR	adenosine deaminase acting on RNA
AI	artificial intelligence
ALS	amyotrophic lateral sclerosis
ALSFRS-R	ALS Functional Rating Scale–Revised
AMT-130	AAV5-miHTT gene therapy AMT-130 for Huntington’s disease
APP	amyloid precursor protein
ASO	antisense oligonucleotide
ASOs	antisense oligonucleotides
AUC	area under the receiver operating characteristic curve
Aβ	amyloid-β peptide
BBB	blood–brain barrier
C9ORF72	chromosome 9 open reading frame 72
CMA	chaperone-mediated autophagy
CNS	central nervous system
CRISPR	clustered regularly interspaced short palindromic repeats
CRISPRi	CRISPR interference
CSF	cerebrospinal fluid
ddPCR	droplet digital polymerase chain reaction
DNA	deoxyribonucleic acid
EV	extracellular vesicle
EVs	extracellular vesicles
FTD	frontotemporal dementia
GPCR	G protein–coupled receptor
HD	Huntington’s disease
HTT	huntingtin
ICV	intracerebroventricular
ISR	integrated stress response
IT	intrathecal
LAMP2A	lysosome-associated membrane protein 2A
LLPS	liquid–liquid phase separation
LTP	long-term potentiation
MAPT	microtubule-associated protein tau
MAPTRx	tau-targeting antisense oligonucleotide MAPT(Rx)
MISEV2023	Minimal Information for Studies of Extracellular Vesicles 2023
MRI	magnetic resonance imaging
NDD	neurodegenerative disease
NDDs	neurodegenerative diseases
NDEVs	neuron-derived extracellular vesicles
NfL	neurofilament light chain
NGS	next-generation sequencing
NMDA	N-methyl-D-aspartate
NRI	net reclassification index
PD	Parkinson’s disease
PET	positron emission tomography
PIWI	P-element-induced wimpy testis protein family
PSP	progressive supranuclear palsy
QC	quality control
RAN	repeat-associated non-ATG
RBP	RNA-binding protein
RBPs	RNA-binding proteins
RISC	RNA-induced silencing complex
RNA	ribonucleic acid
RNAi	RNA interference
RVG	rabies virus glycoprotein
SNP	single-nucleotide polymorphism
SNPs	single-nucleotide polymorphisms
SNCA	alpha-synuclein
SOD1-ALS	amyotrophic lateral sclerosis with SOD1 mutations
TfR1	transferrin receptor 1
XAI	explainable artificial intelligence
ceRNA	competing endogenous RNA
circRNAs	circular RNAs
iLTP	inhibitory long-term potentiation
lncRNA	long non-coding RNA
lncRNAs	long non-coding RNAs
m(6)A	N6-methyladenosine
m6A	N6-methyladenosine
mHTT	mutant huntingtin
miRNA	microRNA
miRNAs	microRNAs
mRNA	messenger RNA
ncRNA	non-coding RNA
ncRNAs	non-coding RNAs
nt	nucleotide
piRNA	PIWI-interacting RNA
piRNAs	PIWI-interacting RNAs
pre-miRNAs	precursor microRNAs
pri-miRNAs	primary microRNAs
qPCR	quantitative polymerase chain reaction

## References

[1] Alzheimer’s Association (2024). 2024 Alzheimer’s disease facts and figures. Alzheimer’s Dement..

[2] Steinmetz J.D., Seeher K.M., Schiess N., Nichols E., Cao B., Servili C., Cavallera V., Cousin E., Hagins H., Moberg M.E. (2024). Global, regional, and national burden of disorders affecting the nervous system, 1990–2021: A systematic analysis for the Global Burden of Disease Study 2021. Lancet Neurol..

[3] Li Y., Sun S. (2025). RNA dysregulation in neurodegenerative diseases. EMBO J..

[4] Alzarea S.I. (2025). Non-coding RNA-mediated gene regulation in Alzheimer’s disease pathogenesis: Molecular insights and emerging innovations. Saudi Pharm. J..

[5] Anilkumar A.K., Vij P., Lopez S., Leslie S.M., Doxtater K., Khan M.M., Yallapu M.M., Chauhan S.C., Maestre G.E., Tripathi M.K. (2024). Long Non-Coding RNAs: New Insights in Neurodegenerative Diseases. Int. J. Mol. Sci..

[6] Ilieva M.S. (2024). Non-Coding RNAs in Neurological and Neuropsychiatric Disorders: Unraveling the Hidden Players in Disease Pathogenesis. Cells.

[7] Talebi Taheri A., Golshadi Z., Zare H., Alinaghipour A., Faghihi Z., Dadgostar E., Tamtaji Z., Aschner M., Mirzaei H., Tamtaji O.R. (2024). The Potential of Targeting Autophagy-Related Non-coding RNAs in the Treatment of Alzheimer’s and Parkinson’s Diseases. Cell. Mol. Neurobiol..

[8] Li J., Wang X. (2024). Functional roles of conserved lncRNAs and circRNAs in eukaryotes. Noncoding RNA Res..

[9] Song G., Yang Z., Guo J., Zheng Y., Su X., Wang X. (2020). Interactions Among lncRNAs/circRNAs, miRNAs, and mRNAs in Neuropathic Pain. Neurotherapeutics.

[10] Li M.L., Wang W., Jin Z.B. (2021). Circular RNAs in the Central Nervous System. Front. Mol. Biosci..

[11] Rajasethupathy P., Antonov I., Sheridan R., Frey S., Sander C., Tuschl T., Kandel E.R. (2012). A role for neuronal piRNAs in the epigenetic control of memory-related synaptic plasticity. Cell.

[12] Jadhav S.P. (2024). MicroRNAs in microglia: Deciphering their role in neurodegenerative diseases. Front. Cell. Neurosci..

[13] Rege S.D., Geetha T., Pondugula S.R., Zizza C.A., Wernette C.M., Babu J.R. (2013). Noncoding RNAs in Neurodegenerative Diseases. ISRN Neurol..

[14] Mo M. (2023). Editorial: Non-coding RNAs in neurodegenerative diseases. Front. Neurosci..

[15] Bartel D.P. (2018). Metazoan MicroRNAs. Cell.

[16] Treiber T., Treiber N., Meister G. (2019). Regulation of microRNA biogenesis and its crosstalk with other cellular pathways. Nat. Rev. Mol. Cell Biol..

[17] Rajgor D., Fiuza M., Parkinson G.T., Hanley J.G. (2017). The PICK1 Ca^2+^ sensor modulates N-methyl-d-aspartate (NMDA) receptor-dependent microRNA-mediated translational repression in neurons. J. Biol. Chem..

[18] Hebert S.S., Papadopoulou A.S., Smith P., Galas M.C., Planel E., Silahtaroglu A.N., Sergeant N., Buee L., De Strooper B. (2010). Genetic ablation of Dicer in adult forebrain neurons results in abnormal tau hyperphosphorylation and neurodegeneration. Hum. Mol. Genet..

[19] Cheng T.L., Wang Z., Liao Q., Zhu Y., Zhou W.H., Xu W., Qiu Z. (2014). MeCP2 suppresses nuclear microRNA processing and dendritic growth by regulating the DGCR8/Drosha complex. Dev. Cell.

[20] Dalla Costa I., Buchanan C.N., Zdradzinski M.D., Sahoo P.K., Smith T.P., Thames E., Kar A.N., Twiss J.L. (2021). The functional organization of axonal mRNA transport and translation. Nat. Rev. Neurosci..

[21] Rajman M., Schratt G. (2017). MicroRNAs in neural development: From master regulators to fine-tuners. Development.

[22] Sotoudeh Anvari M., Vasei H., Najmabadi H., Badv R.S., Golipour A., Mohammadi-Yeganeh S., Salehi S., Mohamadi M., Goodarzynejad H., Mowla S.J. (2022). Identification of microRNAs associated with human fragile X syndrome using next-generation sequencing. Sci. Rep..

[23] Lopez-Gonzalez M.J., Landry M., Favereaux A. (2017). MicroRNA and chronic pain: From mechanisms to therapeutic potential. Pharmacol. Ther..

[24] Zhao Y.Y., Wu Z.J., Zhu L.J., Niu T.X., Liu B., Li J. (2023). Emerging roles of miRNAs in neuropathic pain: From new findings to novel mechanisms. Front. Mol. Neurosci..

[25] Dubes S., Favereaux A., Thoumine O., Letellier M. (2019). miRNA-Dependent Control of Homeostatic Plasticity in Neurons. Front. Cell. Neurosci..

[26] Dubes S., Soula A., Benquet S., Tessier B., Poujol C., Favereaux A., Thoumine O., Letellier M. (2022). miR-124-dependent tagging of synapses by synaptopodin enables input-specific homeostatic plasticity. EMBO J..

[27] Aslani M., Mortazavi-Jahromi S.S., Mirshafiey A. (2021). Efficient roles of miR-146a in cellular and molecular mechanisms of neuroinflammatory disorders: An effectual review in neuroimmunology. Immunol. Lett..

[28] Fan W., Liang C., Ou M., Zou T., Sun F., Zhou H., Cui L. (2020). MicroRNA-146a Is a Wide-Reaching Neuroinflammatory Regulator and Potential Treatment Target in Neurological Diseases. Front. Mol. Neurosci..

[29] Barbosa M., Gomes C., Sequeira C., Goncalves-Ribeiro J., Pina C.C., Carvalho L.A., Moreira R., Vaz S.H., Vaz A.R., Brites D. (2021). Recovery of Depleted miR-146a in ALS Cortical Astrocytes Reverts Cell Aberrancies and Prevents Paracrine Pathogenicity on Microglia and Motor Neurons. Front. Cell Dev. Biol..

[30] Long J.M., Ray B., Lahiri D.K. (2012). MicroRNA-153 physiologically inhibits expression of amyloid-beta precursor protein in cultured human fetal brain cells and is dysregulated in a subset of Alzheimer disease patients. J. Biol. Chem..

[31] Liang C., Zhu H., Xu Y., Huang L., Ma C., Deng W., Liu Y., Qin C. (2012). MicroRNA-153 negatively regulates the expression of amyloid precursor protein and amyloid precursor-like protein 2. Brain Res..

[32] Salmena L., Poliseno L., Tay Y., Kats L., Pandolfi P.P. (2011). A ceRNA hypothesis: The Rosetta Stone of a hidden RNA language?. Cell.

[33] Statello L., Guo C.J., Chen L.L., Huarte M. (2021). Gene regulation by long non-coding RNAs and its biological functions. Nat. Rev. Mol. Cell Biol..

[34] Yamazaki T., Souquere S., Chujo T., Kobelke S., Chong Y.S., Fox A.H., Bond C.S., Nakagawa S., Pierron G., Hirose T. (2018). Functional Domains of NEAT1 Architectural lncRNA Induce Paraspeckle Assembly through Phase Separation. Mol. Cell.

[35] Lellahi S.M., Rosenlund I.A., Hedberg A., Kiaer L.T., Mikkola I., Knutsen E., Perander M. (2018). The long noncoding RNA NEAT1 and nuclear paraspeckles are up-regulated by the transcription factor HSF1 in the heat shock response. J. Biol. Chem..

[36] Sunwoo J.S., Lee S.T., Im W., Lee M., Byun J.I., Jung K.H., Park K.I., Jung K.Y., Lee S.K., Chu K. (2017). Altered Expression of the Long Noncoding RNA NEAT1 in Huntington’s Disease. Mol. Neurobiol..

[37] Yadav M., Harding R.J., Li T., Xu X., Gall-Duncan T., Khan M., Bardile C.F., Sequiera G.L., Duan S., Chandrasekaran R. (2024). Huntingtin is an RNA binding protein and participates in NEAT1-mediated paraspeckles. Sci. Adv..

[38] Taiana E., Ronchetti D., Todoerti K., Nobili L., Tassone P., Amodio N., Neri A. (2020). LncRNA NEAT1 in Paraspeckles: A Structural Scaffold for Cellular DNA Damage Response Systems?. Non-Coding RNA.

[39] Li K., Wang Z. (2023). lncRNA NEAT1: Key player in neurodegenerative diseases. Ageing Res. Rev..

[40] Xie S.P., Zhou F., Li J., Duan S.J. (2019). NEAT1 regulates MPP^+^-induced neuronal injury by targeting miR-124 in neuroblastoma cells. Neurosci. Lett..

[41] Esmaeili A., Yazdanpanah N., Rezaei N. (2025). LncRNAs Orchestrating Neuroinflammation: A Comprehensive Review. Cell. Mol. Neurobiol..

[42] Li F., Wang Y., Yang H., Xu Y., Zhou X., Zhang X., Xie Z., Bi J. (2019). The effect of BACE1-AS on beta-amyloid generation by regulating BACE1 mRNA expression. BMC Mol. Biol..

[43] Sayad A., Najafi S., Hussen B.M., Abdullah S.T., Movahedpour A., Taheri M., Hajiesmaeili M. (2022). The Emerging Roles of the beta-Secretase BACE1 and the Long Non-coding RNA BACE1-AS in Human Diseases: A Focus on Neurodegenerative Diseases and Cancer. Front. Aging Neurosci..

[44] Rani N., Nowakowski T.J., Zhou H., Godshalk S.E., Lisi V., Kriegstein A.R., Kosik K.S. (2016). A Primate lncRNA Mediates Notch Signaling during Neuronal Development by Sequestering miRNA. Neuron.

[45] Wang C., Duan Y., Duan G., Wang Q., Zhang K., Deng X., Qian B., Gu J., Ma Z., Zhang S. (2020). Stress Induces Dynamic, Cytotoxicity-Antagonizing TDP-43 Nuclear Bodies via Paraspeckle LncRNA NEAT1-Mediated Liquid-Liquid Phase Separation. Mol. Cell.

[46] Shelkovnikova T.A., Kukharsky M.S., An H., Dimasi P., Alexeeva S., Shabir O., Heath P.R., Buchman V.L. (2018). Protective paraspeckle hyper-assembly downstream of TDP-43 loss of function in amyotrophic lateral sclerosis. Mol. Neurodegener..

[47] Zhao Y., Wang Z., Mao Y., Li B., Zhu Y., Zhang S., Wang S., Jiang Y., Xu N., Xie Y. (2020). NEAT1 regulates microtubule stabilization via FZD3/GSK3beta/P-tau pathway in SH-SY5Y cells and APP/PS1 mice. Aging.

[48] Li Y., Fan H., Ni M., Zhang W., Fang F., Sun J., Lyu P., Ma P. (2023). Targeting lncRNA NEAT1 Hampers Alzheimer’s Disease Progression. Neuroscience.

[49] Bai Y., Yao H.H. (2023). Circular RNAs: Diagnostic and Therapeutic Perspectives in CNS Diseases. Curr. Med. Sci..

[50] Jeck W.R., Sorrentino J.A., Wang K., Slevin M.K., Burd C.E., Liu J., Marzluff W.F., Sharpless N.E. (2013). Circular RNAs are abundant, conserved, and associated with ALU repeats. RNA.

[51] Rybak-Wolf A., Stottmeister C., Glazar P., Jens M., Pino N., Giusti S., Hanan M., Behm M., Bartok O., Ashwal-Fluss R. (2015). Circular RNAs in the Mammalian Brain Are Highly Abundant, Conserved, and Dynamically Expressed. Mol. Cell.

[52] Westholm J.O., Miura P., Olson S., Shenker S., Joseph B., Sanfilippo P., Celniker S.E., Graveley B.R., Lai E.C. (2014). Genome-wide analysis of drosophila circular RNAs reveals their structural and sequence properties and age-dependent neural accumulation. Cell Rep..

[53] Gruner H., Cortes-Lopez M., Cooper D.A., Bauer M., Miura P. (2016). CircRNA accumulation in the aging mouse brain. Sci. Rep..

[54] Zhou W.Y., Cai Z.R., Liu J., Wang D.S., Ju H.Q., Xu R.H. (2020). Circular RNA: Metabolism, functions and interactions with proteins. Mol. Cancer.

[55] Hansen T.B., Jensen T.I., Clausen B.H., Bramsen J.B., Finsen B., Damgaard C.K., Kjems J. (2013). Natural RNA circles function as efficient microRNA sponges. Nature.

[56] Memczak S., Jens M., Elefsinioti A., Torti F., Krueger J., Rybak A., Maier L., Mackowiak S.D., Gregersen L.H., Munschauer M. (2013). Circular RNAs are a large class of animal RNAs with regulatory potency. Nature.

[57] Piwecka M., Glazar P., Hernandez-Miranda L.R., Memczak S., Wolf S.A., Rybak-Wolf A., Filipchyk A., Klironomos F., Cerda Jara C.A., Fenske P. (2017). Loss of a mammalian circular RNA locus causes miRNA deregulation and affects brain function. Science.

[58] Du W.W., Yang W., Liu E., Yang Z., Dhaliwal P., Yang B.B. (2016). Foxo3 circular RNA retards cell cycle progression via forming ternary complexes with p21 and CDK2. Nucleic Acids Res..

[59] Zajaczkowski E.L., Bredy T.W. (2021). Circular RNAs in the Brain: A Possible Role in Memory?. Neuroscientist.

[60] Zajaczkowski E.L., Zhao Q., Liau W.S., Gong H., Madugalle S.U., Periyakaruppiah A., Leighton L.J., Musgrove M., Ren H., Davies J. (2023). Localised Cdr1as activity is required for fear extinction memory. Neurobiol. Learn. Mem..

[61] Ozata D.M., Gainetdinov I., Zoch A., O’Carroll D., Zamore P.D. (2019). PIWI-interacting RNAs: Small RNAs with big functions. Nat. Rev. Genet..

[62] Loubalova Z., Konstantinidou P., Haase A.D. (2023). Themes and variations on piRNA-guided transposon control. Mob. DNA.

[63] Andersen P.R., Tirian L., Vunjak M., Brennecke J. (2017). A heterochromatin-dependent transcription machinery drives piRNA expression. Nature.

[64] Li Z., Tang X., Shen E.Z. (2020). How mammalian piRNAs instruct de novo DNA methylation of transposons. Signal Transduct. Target. Ther..

[65] Sun W., Samimi H., Gamez M., Zare H., Frost B. (2018). Pathogenic tau-induced piRNA depletion promotes neuronal death through transposable element dysregulation in neurodegenerative tauopathies. Nat. Neurosci..

[66] Guo C., Jeong H.H., Hsieh Y.C., Klein H.U., Bennett D.A., De Jager P.L., Liu Z., Shulman J.M. (2018). Tau Activates Transposable Elements in Alzheimer’s Disease. Cell Rep..

[67] Ochoa E., Ramirez P., Gonzalez E., De Mange J., Ray W.J., Bieniek K.F., Frost B. (2023). Pathogenic tau-induced transposable element-derived dsRNA drives neuroinflammation. Sci. Adv..

[68] Gorbunova V., Seluanov A., Mita P., McKerrow W., Fenyo D., Boeke J.D., Linker S.B., Gage F.H., Kreiling J.A., Petrashen A.P. (2021). The role of retrotransposable elements in ageing and age-associated diseases. Nature.

[69] Abdelhamid R.F., Ogawa K., Beck G., Ikenaka K., Takeuchi E., Yasumizu Y., Jinno J., Kimura Y., Baba K., Nagai Y. (2022). piRNA/PIWI Protein Complex as a Potential Biomarker in Sporadic Amyotrophic Lateral Sclerosis. Mol. Neurobiol..

[70] Kim K.W., Tang N.H., Andrusiak M.G., Wu Z., Chisholm A.D., Jin Y. (2018). A Neuronal piRNA Pathway Inhibits Axon Regeneration in *C. elegans*. Neuron.

[71] Kawahara Y., Mieda-Sato A. (2012). TDP-43 promotes microRNA biogenesis as a component of the Drosha and Dicer complexes. Proc. Natl. Acad. Sci. USA.

[72] Morlando M., Dini Modigliani S., Torrelli G., Rosa A., Di Carlo V., Caffarelli E., Bozzoni I. (2012). FUS stimulates microRNA biogenesis by facilitating co-transcriptional Drosha recruitment. EMBO J..

[73] Kim H.J., Taylor J.P. (2017). Lost in Transportation: Nucleocytoplasmic Transport Defects in ALS and Other Neurodegenerative Diseases. Neuron.

[74] Rosenthal J.J., Seeburg P.H. (2012). A-to-I RNA editing: Effects on proteins key to neural excitability. Neuron.

[75] Zaccara S., Ries R.J., Jaffrey S.R. (2019). Reading, writing and erasing mRNA methylation. Nat. Rev. Mol. Cell Biol..

[76] Ries R.J., Pickering B.F., Poh H.X., Namkoong S., Jaffrey S.R. (2023). m^6^A governs length-dependent enrichment of mRNAs in stress granules. Nat. Struct. Mol. Biol..

[77] Miller T.M., Cudkowicz M.E., Genge A., Shaw P.J., Sobue G., Bucelli R.C., Chio A., Van Damme P., Ludolph A.C., Glass J.D. (2022). Trial of Antisense Oligonucleotide Tofersen for SOD1 ALS. N. Engl. J. Med..

[78] Zong Y., Wang H., Dong W., Quan X., Zhu H., Xu Y., Huang L., Ma C., Qin C. (2011). miR-29c regulates BACE1 protein expression. Brain Res..

[79] Lei X., Lei L., Zhang Z., Zhang Z., Cheng Y. (2015). Downregulated miR-29c correlates with increased BACE1 expression in sporadic Alzheimer’s disease. Int. J. Clin. Exp. Pathol..

[80] Liu Z., Zhang H., Sun L., Zhu K., Lang W. (2020). miR-29c-3p Increases Cell Viability and Suppresses Apoptosis by Regulating the TNFAIP1/NF-kappaB Signaling Pathway via TNFAIP1 in Abeta-Treated Neuroblastoma Cells. Neurochem. Res..

[81] El Fatimy R., Li S., Chen Z., Mushannen T., Gongala S., Wei Z., Balu D.T., Rabinovsky R., Cantlon A., Elkhal A. (2018). MicroRNA-132 provides neuroprotection for tauopathies via multiple signaling pathways. Acta Neuropathol..

[82] Walgrave H., Balusu S., Snoeck S., Vanden Eynden E., Craessaerts K., Thrupp N., Wolfs L., Horre K., Fourne Y., Ronisz A. (2021). Restoring miR-132 expression rescues adult hippocampal neurogenesis and memory deficits in Alzheimer’s disease. Cell Stem Cell.

[83] Wong H.K., Veremeyko T., Patel N., Lemere C.A., Walsh D.M., Esau C., Vanderburg C., Krichevsky A.M. (2013). De-repression of FOXO3a death axis by microRNA-132 and -212 causes neuronal apoptosis in Alzheimer’s disease. Hum. Mol. Genet..

[84] Bazrgar M., Khodabakhsh P., Prudencio M., Mohagheghi F., Ahmadiani A. (2021). The role of microRNA-34 family in Alzheimer’s disease: A potential molecular link between neurodegeneration and metabolic disorders. Pharmacol. Res..

[85] Dong Z., Gu H., Guo Q., Liang S., Xue J., Yao F., Liu X., Li F., Liu H., Sun L. (2021). Profiling of Serum Exosome MiRNA Reveals the Potential of a MiRNA Panel as Diagnostic Biomarker for Alzheimer’s Disease. Mol. Neurobiol..

[86] Song S., Lee J.U., Jeon M.J., Kim S., Sim S.J. (2022). Detection of multiplex exosomal miRNAs for clinically accurate diagnosis of Alzheimer’s disease using label-free plasmonic biosensor based on DNA-Assembled advanced plasmonic architecture. Biosens. Bioelectron..

[87] Alhenaky A., Alhazmi S., Alamri S.H., Alkhatabi H.A., Alharthi A., Alsaleem M.A., Abdelnour S.A., Hassan S.M. (2024). Exosomal MicroRNAs in Alzheimer’s Disease: Unveiling Their Role and Pioneering Tools for Diagnosis and Treatment. J. Clin. Med..

[88] Yu X., Sun X., Wei M., Deng S., Zhang Q., Guo T., Shao K., Zhang M., Jiang J., Han Y. (2024). Innovative Multivariable Model Combining MRI Radiomics and Plasma Indexes Predicts Alzheimer’s Disease Conversion: Evidence from a 2-Cohort Longitudinal Study. Research.

[89] Wang Y., Chen H.J., Cheng Y., Xie Y., Cheng Y., Zhao S., Jiang Y., Bai T., Huo Y., Wang K. (2025). Multimodal integration of plasma biomarkers, MRI, and genetic risk to predict cerebral amyloid burden in Alzheimer’s disease. Neuroimage.

[90] Gascon E., Gao F.B. (2012). Cause or Effect: Misregulation of microRNA Pathways in Neurodegeneration. Front. Neurosci..

[91] Shaheen N., Shaheen A., Osama M., Nashwan A.J., Bharmauria V., Flouty O. (2024). MicroRNAs regulation in Parkinson’s disease, and their potential role as diagnostic and therapeutic targets. NPJ Park. Dis..

[92] Henrich M.T., Oertel W.H., Surmeier D.J., Geibl F.F. (2023). Mitochondrial dysfunction in Parkinson’s disease—A key disease hallmark with therapeutic potential. Mol. Neurodegener..

[93] McMillan K.J., Murray T.K., Bengoa-Vergniory N., Cordero-Llana O., Cooper J., Buckley A., Wade-Martins R., Uney J.B., O’Neill M.J., Wong L.F. (2017). Loss of MicroRNA-7 Regulation Leads to alpha-Synuclein Accumulation and Dopaminergic Neuronal Loss In Vivo. Mol. Ther..

[94] Sala G., Marinig D., Arosio A., Ferrarese C. (2016). Role of Chaperone-Mediated Autophagy Dysfunctions in the Pathogenesis of Parkinson’s Disease. Front. Mol. Neurosci..

[95] Sang Q., Liu X., Wang L., Qi L., Sun W., Wang W., Sun Y., Zhang H. (2018). CircSNCA downregulation by pramipexole treatment mediates cell apoptosis and autophagy in Parkinson’s disease by targeting miR-7. Aging.

[96] Liao J., Zhang Q., Huang J., He H., Lei J., Shen Y., Wang J., Xiao Y. (2023). The emerging role of circular RNAs in Parkinson’s disease. Front. Neurosci..

[97] Minones-Moyano E., Porta S., Escaramis G., Rabionet R., Iraola S., Kagerbauer B., Espinosa-Parrilla Y., Ferrer I., Estivill X., Marti E. (2011). MicroRNA profiling of Parkinson’s disease brains identifies early downregulation of miR-34b/c which modulate mitochondrial function. Hum. Mol. Genet..

[98] Citterio L.A., Mancuso R., Agostini S., Meloni M., Clerici M. (2023). Serum and Exosomal miR-7-1-5p and miR-223-3p as Possible Biomarkers for Parkinson’s Disease. Biomolecules.

[99] Vaitkiene P., Pranckeviciene A., Radziunas A., Miseikaite A., Miniotaite G., Belickiene V., Laucius O., Deltuva V. (2024). Association of Serum Extracellular Vesicle miRNAs with Cognitive Functioning and Quality of Life in Parkinson’s Disease. Biomolecules.

[100] Dos Santos M.C.T., Barreto-Sanz M.A., Correia B.R.S., Bell R., Widnall C., Perez L.T., Berteau C., Schulte C., Scheller D., Berg D. (2018). miRNA-based signatures in cerebrospinal fluid as potential diagnostic tools for early stage Parkinson’s disease. Oncotarget.

[101] Wu L., Xu Q., Zhou M., Chen Y., Jiang C., Jiang Y., Lin Y., He Q., Zhao L., Dong Y. (2022). Plasma miR-153 and miR-223 Levels as Potential Biomarkers in Parkinson’s Disease. Front. Neurosci..

[102] Zhang J., Zhao M., Yan R., Liu J., Maddila S., Junn E., Mouradian M.M. (2021). MicroRNA-7 Protects Against Neurodegeneration Induced by α-Synuclein Preformed Fibrils in the Mouse Brain. Neurotherapeutics.

[103] Azadfar P., Noormohammadi Z., Noroozian M., Eidi A., Mortazavi P. (2020). Effect of memantine on expression of Bace1-as and Bace1 genes in STZ-induced Alzheimeric rats. Mol. Biol. Rep..

[104] Choi D.C., Yoo M., Kabaria S., Junn E. (2018). MicroRNA-7 facilitates the degradation of alpha-synuclein and its aggregates by promoting autophagy. Neurosci. Lett..

[105] Fan Z., Lu M., Qiao C., Zhou Y., Ding J.H., Hu G. (2016). MicroRNA-7 Enhances Subventricular Zone Neurogenesis by Inhibiting NLRP3/Caspase-1 Axis in Adult Neural Stem Cells. Mol. Neurobiol..

[106] Bates G.P., Dorsey R., Gusella J.F., Hayden M.R., Kay C., Leavitt B.R., Nance M., Ross C.A., Scahill R.I., Wetzel R. (2015). Huntington disease. Nat. Rev. Dis. Primers.

[107] de Mezer M., Wojciechowska M., Napierala M., Sobczak K., Krzyzosiak W.J. (2011). Mutant CAG repeats of Huntingtin transcript fold into hairpins, form nuclear foci and are targets for RNA interference. Nucleic Acids Res..

[108] Nalavade R., Griesche N., Ryan D.P., Hildebrand S., Krauss S. (2013). Mechanisms of RNA-induced toxicity in CAG repeat disorders. Cell Death Dis..

[109] Ayyildiz D., Bergonzoni G., Monziani A., Tripathi T., Doring J., Kerschbamer E., Di Leva F., Pennati E., Donini L., Kovalenko M. (2023). CAG repeat expansion in the Huntington’s disease gene shapes linear and circular RNAs biogenesis. PLoS Genet..

[110] Sonmez A., Mustafa R., Ryll S.T., Tuorto F., Wacheul L., Ponti D., Litke C., Hering T., Kojer K., Koch J. (2021). Nucleolar stress controls mutant Huntington toxicity and monitors Huntington’s disease progression. Cell Death Dis..

[111] Aviner R., Lee T.T., Masto V.B., Li K.H., Andino R., Frydman J. (2024). Polyglutamine-mediated ribotoxicity disrupts proteostasis and stress responses in Huntington’s disease. Nat. Cell Biol..

[112] Tabrizi S.J., Leavitt B.R., Landwehrmeyer G.B., Wild E.J., Saft C., Barker R.A., Blair N.F., Craufurd D., Priller J., Rickards H. (2019). Targeting Huntingtin Expression in Patients with Huntington’s Disease. N. Engl. J. Med..

[113] Conroy F., Miller R., Alterman J.F., Hassler M.R., Echeverria D., Godinho B., Knox E.G., Sapp E., Sousa J., Yamada K. (2022). Chemical engineering of therapeutic siRNAs for allele-specific gene silencing in Huntington’s disease models. Nat. Commun..

[114] Hirunagi T., Sahashi K., Tachikawa K., Leu A.I., Nguyen M., Mukthavaram R., Karmali P.P., Chivukula P., Tohnai G., Iida M. (2021). Selective suppression of polyglutamine-expanded protein by lipid nanoparticle-delivered siRNA targeting CAG expansions in the mouse CNS. Mol. Ther. Nucleic Acids.

[115] Keskin S., Brouwers C.C., Sogorb-Gonzalez M., Martier R., Depla J.A., Valles A., van Deventer S.J., Konstantinova P., Evers M.M. (2019). AAV5-miHTT Lowers Huntingtin mRNA and Protein without Off-Target Effects in Patient-Derived Neuronal Cultures and Astrocytes. Mol. Ther. Methods Clin. Dev..

[116] Cheng P.H., Li C.L., Chang Y.F., Tsai S.J., Lai Y.Y., Chan A.W., Chen C.M., Yang S.H. (2013). miR-196a ameliorates phenotypes of Huntington disease in cell, transgenic mouse, and induced pluripotent stem cell models. Am. J. Hum. Genet..

[117] Kunkanjanawan T., Carter R.L., Prucha M.S., Yang J., Parnpai R., Chan A.W. (2016). miR-196a Ameliorates Cytotoxicity and Cellular Phenotype in Transgenic Huntington’s Disease Monkey Neural Cells. PLoS ONE.

[118] Hawley Z.C.E., Campos-Melo D., Strong M.J. (2020). Evidence of A Negative Feedback Network Between TDP-43 and miRNAs Dependent on TDP-43 Nuclear Localization. J. Mol. Biol..

[119] Hawley Z.C.E., Campos-Melo D., Strong M.J. (2019). MiR-105 and miR-9 regulate the mRNA stability of neuronal intermediate filaments. Implications for the pathogenesis of amyotrophic lateral sclerosis (ALS). Brain Res..

[120] Williams A.H., Valdez G., Moresi V., Qi X., McAnally J., Elliott J.L., Bassel-Duby R., Sanes J.R., Olson E.N. (2009). MicroRNA-206 delays ALS progression and promotes regeneration of neuromuscular synapses in mice. Science.

[121] Waller R., Goodall E.F., Milo M., Cooper-Knock J., Da Costa M., Hobson E., Kazoka M., Wollff H., Heath P.R., Shaw P.J. (2017). Serum miRNAs miR-206, 143–3p and 374b-5p as potential biomarkers for amyotrophic lateral sclerosis (ALS). Neurobiol. Aging.

[122] Ash P.E., Bieniek K.F., Gendron T.F., Caulfield T., Lin W.L., Dejesus-Hernandez M., van Blitterswijk M.M., Jansen-West K., Paul J.W., Rademakers R. (2013). Unconventional translation of C9ORF72 GGGGCC expansion generates insoluble polypeptides specific to c9FTD/ALS. Neuron.

[123] Cooper-Knock J., Walsh M.J., Higginbottom A., Robin Highley J., Dickman M.J., Edbauer D., Ince P.G., Wharton S.B., Wilson S.A., Kirby J. (2014). Sequestration of multiple RNA recognition motif-containing proteins by C9orf72 repeat expansions. Brain.

[124] Cooper-Knock J., Bury J.J., Heath P.R., Wyles M., Higginbottom A., Gelsthorpe C., Highley J.R., Hautbergue G., Rattray M., Kirby J. (2015). C9ORF72 GGGGCC Expanded Repeats Produce Splicing Dysregulation which Correlates with Disease Severity in Amyotrophic Lateral Sclerosis. PLoS ONE.

[125] Parameswaran J., Zhang N., Braems E., Tilahun K., Pant D.C., Yin K., Asress S., Heeren K., Banerjee A., Davis E. (2023). Antisense, but not sense, repeat expanded RNAs activate PKR/eIF2alpha-dependent ISR in C9ORF72 FTD/ALS. Elife.

[126] Smith P.Y., Hernandez-Rapp J., Jolivette F., Lecours C., Bisht K., Goupil C., Dorval V., Parsi S., Morin F., Planel E. (2015). miR-132/212 deficiency impairs tau metabolism and promotes pathological aggregation in vivo. Hum. Mol. Genet..

[127] Donnelly C.J., Zhang P.W., Pham J.T., Haeusler A.R., Mistry N.A., Vidensky S., Daley E.L., Poth E.M., Hoover B., Fines D.M. (2013). RNA toxicity from the ALS/FTD C9ORF72 expansion is mitigated by antisense intervention. Neuron.

[128] Sareen D., O’Rourke J.G., Meera P., Muhammad A.K., Grant S., Simpkinson M., Bell S., Carmona S., Ornelas L., Sahabian A. (2013). Targeting RNA foci in iPSC-derived motor neurons from ALS patients with a C9ORF72 repeat expansion. Sci. Transl. Med..

[129] Basso D., Padoan A., Laufer T., Aneloni V., Moz S., Schroers H., Pelloso M., Saiz A., Krapp M., Fogar P. (2017). Relevance of pre-analytical blood management on the emerging cardiovascular protein biomarkers TWEAK and HMGB1 and on miRNA serum and plasma profiling. Clin. Biochem..

[130] Shah J.S., Soon P.S., Marsh D.J. (2016). Comparison of Methodologies to Detect Low Levels of Hemolysis in Serum for Accurate Assessment of Serum microRNAs. PLoS ONE.

[131] Sorensen S.S., Nygaard A.B., Christensen T. (2016). miRNA expression profiles in cerebrospinal fluid and blood of patients with Alzheimer’s disease and other types of dementia—An exploratory study. Transl. Neurodegener..

[132] Welsh J.A., Goberdhan D.C.I., O’Driscoll L., Buzas E.I., Blenkiron C., Bussolati B., Cai H., Di Vizio D., Driedonks T.A.P., Erdbrugger U. (2024). Minimal information for studies of extracellular vesicles (MISEV2023): From basic to advanced approaches. J. Extracell. Vesicles.

[133] Norman M., Ter-Ovanesyan D., Trieu W., Lazarovits R., Kowal E.J.K., Lee J.H., Chen-Plotkin A.S., Regev A., Church G.M., Walt D.R. (2021). L1CAM is not associated with extracellular vesicles in human cerebrospinal fluid or plasma. Nat. Methods.

[134] Fotuhi S.N., Khalaj-Kondori M., Hoseinpour Feizi M.A., Talebi M. (2019). Long Non-coding RNA BACE1-AS May Serve as an Alzheimer’s Disease Blood-Based Biomarker. J. Mol. Neurosci..

[135] Khodayi M., Khalaj-Kondori M., Hoseinpour Feizi M.A., Jabarpour Bonyadi M., Talebi M. (2022). Plasma lncRNA profiling identified BC200 and NEAT1 lncRNAs as potential blood-based biomarkers for late-onset Alzheimer’s disease. EXCLI J..

[136] Ma W., Li Y., Wang C., Xu F., Wang M., Liu Y. (2016). Serum miR-221 serves as a biomarker for Parkinson’s disease. Cell Biochem. Funct..

[137] Schulz J., Takousis P., Wohlers I., Itua I.O.G., Dobricic V., Rucker G., Binder H., Middleton L., Ioannidis J.P.A., Perneczky R. (2019). Meta-analyses identify differentially expressed micrornas in Parkinson’s disease. Ann. Neurol..

[138] Chunhui G., Yanqiu Y., Jibing C., Ning L., Fujun L. (2025). Exosomes and non-coding RNAs: Bridging the gap in Alzheimer’s pathogenesis and therapeutics. Metab. Brain Dis..

[139] Feng L., Liao Y.T., He J.C., Xie C.L., Chen S.Y., Fan H.H., Su Z.P., Wang Z. (2018). Plasma long non-coding RNA BACE1 as a novel biomarker for diagnosis of Alzheimer disease. BMC Neurol..

[140] Soliman H.M., Ghonaim G.A., Gharib S.M., Chopra H., Farag A.K., Hassanin M.H., Nagah A., Emad-Eldin M., Hashem N.E., Yahya G. (2021). Exosomes in Alzheimer’s Disease: From Being Pathological Players to Potential Diagnostics and Therapeutics. Int. J. Mol. Sci..

[141] Gutierrez-Tordera L., Papandreou C., Novau-Ferre N., Garcia-Gonzalez P., Rojas M., Marquie M., Chapado L.A., Papagiannopoulos C., Fernandez-Castillo N., Valero S. (2024). Exploring small non-coding RNAs as blood-based biomarkers to predict Alzheimer’s disease. Cell Biosci..

[142] Park C., Weerakkody J.S., Schneider R., Miao S., Pitt D. (2024). CNS cell-derived exosome signatures as blood-based biomarkers of neurodegenerative diseases. Front. Neurosci..

[143] Thery C., Witwer K.W., Aikawa E. (2018). Minimal information for studies of extracellular vesicles 2018 (MISEV2018): A position statement of the International Society for Extracellular Vesicles and update of the MISEV2014 guidelines. J. Extracell. Vesicles.

[144] Kirschner M.B., Edelman J.J., Kao S.C., Vallely M.P., van Zandwijk N., Reid G. (2013). The Impact of Hemolysis on Cell-Free microRNA Biomarkers. Front. Genet..

[145] Sidhom K., Obi P.O., Saleem A. (2020). A Review of Exosomal Isolation Methods: Is Size Exclusion Chromatography the Best Option?. Int. J. Mol. Sci..

[146] Xu Y.Z., Cheng M.G., Wang X., Hu Y. (2021). The emerging role of non-coding RNAs from extracellular vesicles in Alzheimer’s disease. J. Integr. Neurosci..

[147] Sun Y.M., Chen Y.Q. (2020). Principles and innovative technologies for decrypting noncoding RNAs: From discovery and functional prediction to clinical application. J. Hematol. Oncol..

[148] Almohaimeed H.M., Assiri R., Althubaiti E.H., Aggad W.S., Shaheen S., Shaheen M.Y., Batarfi M.A., Alharbi N.A., Alshehri A.M., Alkhudhairy B.S.M. (2023). Non-coding RNAs as key players in the neurodegenerative diseases: Multi-platform strategies and approaches for exploring the Genome’s dark matter. J. Chem. Neuroanat..

[149] Mahmud T., Barua K., Habiba S.U., Sharmen N., Hossain M.S., Andersson K. (2024). An Explainable AI Paradigm for Alzheimer’s Diagnosis Using Deep Transfer Learning. Diagnostics.

[150] Shobeiri P., Alilou S., Jaberinezhad M., Zare F., Karimi N., Maleki S., Teixeira A.L., Perry G., Rezaei N. (2023). Circulating long non-coding RNAs as novel diagnostic biomarkers for Alzheimer’s disease (AD): A systematic review and meta-analysis. PLoS ONE.

[151] Hao Y., Xie B., Fu X., Xu R., Yang Y. (2022). New Insights into lncRNAs in Abeta Cascade Hypothesis of Alzheimer’s Disease. Biomolecules.

[152] Zhao M.Y., Wang G.Q., Wang N.N., Yu Q.Y., Liu R.L., Shi W.Q. (2019). The long-non-coding RNA NEAT1 is a novel target for Alzheimer’s disease progression via miR-124/BACE1 axis. Neurol. Res..

[153] An F., Gong G., Wang Y., Bian M., Yu L., Wei C. (2017). MiR-124 acts as a target for Alzheimer’s disease by regulating BACE1. Oncotarget.

[154] Wang F., Liang Y., Wang Q.W. (2024). Interpretable machine learning-driven biomarker identification and validation for Alzheimer’s disease. Sci. Rep..

[155] Bloch L., Friedrich C.M., Alzheimer’s Disease Neuroimaging I. (2021). Data analysis with Shapley values for automatic subject selection in Alzheimer’s disease data sets using interpretable machine learning. Alzheimer’s Res. Ther..

[156] Lin R., Wichadakul D. (2022). Interpretable Deep Learning Model Reveals Subsequences of Various Functions for Long Non-Coding RNA Identification. Front. Genet..

[157] Zhang Y., Zhao Y., Ao X., Yu W., Zhang L., Wang Y., Chang W. (2021). The Role of Non-coding RNAs in Alzheimer’s Disease: From Regulated Mechanism to Therapeutic Targets and Diagnostic Biomarkers. Front. Aging Neurosci..

[158] Wang D., Wang P., Bian X., Xu S., Zhou Q., Zhang Y., Ding M., Han M., Huang L., Bi J. (2020). Elevated plasma levels of exosomal BACE1-AS combined with the volume and thickness of the right entorhinal cortex may serve as a biomarker for the detection of Alzheimer’s disease. Mol. Med. Rep..

[159] Ding H., Huang Z., Chen M., Wang C., Chen X., Chen J., Zhang J. (2016). Identification of a panel of five serum miRNAs as a biomarker for Parkinson’s disease. Park. Relat. Disord..

[160] Ghit A., Deeb H.E. (2022). Cytokines, miRNAs, and Antioxidants as Combined Non-invasive Biomarkers for Parkinson’s Disease. J. Mol. Neurosci..

[161] Vabalas A., Gowen E., Poliakoff E., Casson A.J. (2019). Machine learning algorithm validation with a limited sample size. PLoS ONE.

[162] Shen X., Corey D.R. (2018). Chemistry, mechanism and clinical status of antisense oligonucleotides and duplex RNAs. Nucleic Acids Res..

[163] Bennett C.F., Kordasiewicz H.B., Cleveland D.W. (2021). Antisense Drugs Make Sense for Neurological Diseases. Annu. Rev. Pharmacol. Toxicol..

[164] Mummery C.J., Borjesson-Hanson A., Blackburn D.J., Vijverberg E.G.B., De Deyn P.P., Ducharme S., Jonsson M., Schneider A., Rinne J.O., Ludolph A.C. (2023). Tau-targeting antisense oligonucleotide MAPT(Rx) in mild Alzheimer’s disease: A phase 1b, randomized, placebo-controlled trial. Nat. Med..

[165] Rook M.E., Southwell A.L. (2022). Antisense Oligonucleotide Therapy: From Design to the Huntington Disease Clinic. BioDrugs.

[166] Ramachandran P.S., Keiser M.S., Davidson B.L. (2013). Recent advances in RNA interference therapeutics for CNS diseases. Neurotherapeutics.

[167] Martier R., Konstantinova P. (2020). Gene Therapy for Neurodegenerative Diseases: Slowing Down the Ticking Clock. Front. Neurosci..

[168] Seyhan A.A. (2024). Trials and Tribulations of MicroRNA Therapeutics. Int. J. Mol. Sci..

[169] Zhang M., He P., Bian Z. (2021). Long Noncoding RNAs in Neurodegenerative Diseases: Pathogenesis and Potential Implications as Clinical Biomarkers. Front. Mol. Neurosci..

[170] Liu S.J., Horlbeck M.A., Cho S.W., Birk H.S., Malatesta M., He D., Attenello F.J., Villalta J.E., Cho M.Y., Chen Y. (2017). CRISPRi-based genome-scale identification of functional long noncoding RNA loci in human cells. Science.

[171] Abudayyeh O.O., Gootenberg J.S., Essletzbichler P., Han S., Joung J., Belanto J.J., Verdine V., Cox D.B.T., Kellner M.J., Regev A. (2017). RNA targeting with CRISPR-Cas13. Nature.

[172] Moriyama H., Yokota T. (2024). Recent Progress of Antisense Oligonucleotide Therapy for Superoxide-Dismutase-1-Mutated Amyotrophic Lateral Sclerosis: Focus on Tofersen. Genes.

[173] Miller T., Cudkowicz M., Shaw P.J., Andersen P.M., Atassi N., Bucelli R.C., Genge A., Glass J., Ladha S., Ludolph A.L. (2020). Phase 1-2 Trial of Antisense Oligonucleotide Tofersen for SOD1 ALS. N. Engl. J. Med..

[174] Everett W.H., Bucelli R.C. (2024). Tofersen for SOD1 ALS. Neurodegener. Dis. Manag..

[175] McGuigan A., Blair H.A. (2025). Tofersen: A Review in Amyotrophic Lateral Sclerosis Associated with SOD1 Mutations. CNS Drugs.

[176] McColgan P., Thobhani A., Boak L., Schobel S.A., Nicotra A., Palermo G., Trundell D., Zhou J., Schlegel V., Sanwald Ducray P. (2023). Tominersen in Adults with Manifest Huntington’s Disease. N. Engl. J. Med..

[177] Tabrizi S.J., Estevez-Fraga C., van Roon-Mom W.M.C., Flower M.D., Scahill R.I., Wild E.J., Munoz-Sanjuan I., Sampaio C., Rosser A.E., Leavitt B.R. (2022). Potential disease-modifying therapies for Huntington’s disease: Lessons learned and future opportunities. Lancet Neurol..

[178] Spronck E.A., Valles A., Lampen M.H., Montenegro-Miranda P.S., Keskin S., Heijink L., Evers M.M., Petry H., Deventer S.J.V., Konstantinova P. (2021). Intrastriatal Administration of AAV5-miHTT in Non-Human Primates and Rats Is Well Tolerated and Results in miHTT Transgene Expression in Key Areas of Huntington Disease Pathology. Brain Sci..

[179] Valles A., Evers M.M., Stam A., Sogorb-Gonzalez M., Brouwers C., Vendrell-Tornero C., Acar-Broekmans S., Paerels L., Klima J., Bohuslavova B. (2021). Widespread and sustained target engagement in Huntington’s disease minipigs upon intrastriatal microRNA-based gene therapy. Sci. Transl. Med..

[180] Byun S., Lee M., Kim M. (2022). Gene Therapy for Huntington’s Disease: The Final Strategy for a Cure?. J. Mov. Disord..

[181] Komatsu H. (2021). Innovative Therapeutic Approaches for Huntington’s Disease: From Nucleic Acids to GPCR-Targeting Small Molecules. Front. Cell. Neurosci..

[182] Wu D., Chen Q., Chen X., Han F., Chen Z., Wang Y. (2023). The blood-brain barrier: Structure, regulation, and drug delivery. Signal Transduct. Target. Ther..

[183] Mazur C., Powers B., Zasadny K., Sullivan J.M., Dimant H., Kamme F., Hesterman J., Matson J., Oestergaard M., Seaman M. (2019). Brain pharmacology of intrathecal antisense oligonucleotides revealed through multimodal imaging. JCI Insight.

[184] Johnsen K.B., Bak M., Kempen P.J., Melander F., Burkhart A., Thomsen M.S., Nielsen M.S., Moos T., Andresen T.L. (2018). Antibody affinity and valency impact brain uptake of transferrin receptor-targeted gold nanoparticles. Theranostics.

[185] Pornnoppadol G., Bond L.G., Lucas M.J., Zupancic J.M., Kuo Y.H., Zhang B., Greineder C.F., Tessier P.M. (2024). Bispecific antibody shuttles targeting CD98hc mediate efficient and long-lived brain delivery of IgGs. Cell Chem. Biol..

[186] Kingwell K. (2023). A new shuttle for drug delivery across the blood-brain barrier. Nat. Rev. Drug Discov..

[187] Alvarez-Erviti L., Seow Y., Yin H., Betts C., Lakhal S., Wood M.J. (2011). Delivery of siRNA to the mouse brain by systemic injection of targeted exosomes. Nat. Biotechnol..

[188] Herrmann I.K., Wood M.J.A., Fuhrmann G. (2021). Extracellular vesicles as a next-generation drug delivery platform. Nat. Nanotechnol..

[189] Kimiz-Gebologlu I., Oncel S.S. (2022). Exosomes: Large-scale production, isolation, drug loading efficiency, and biodistribution and uptake. J. Control. Release.

[190] Cooper J.M., Wiklander P.B., Nordin J.Z., Al-Shawi R., Wood M.J., Vithlani M., Schapira A.H., Simons J.P., El-Andaloussi S., Alvarez-Erviti L. (2014). Systemic exosomal siRNA delivery reduced alpha-synuclein aggregates in brains of transgenic mice. Mov. Disord..

[191] Sumner C.J., Miller T.M. (2024). The expanding application of antisense oligonucleotides to neurodegenerative diseases. J. Clin. Investig..

[192] Crooke S.T., Baker B.F., Crooke R.M., Liang X.H. (2021). Antisense technology: An overview and prospectus. Nat. Rev. Drug Discov..

[193] Henry S.P., Arfvidsson C., Arrington J., Canadi J., Crowe D., Gupta S., Lohmann S., Massonnet B., Mytych D., Rogers T. (2022). Assessment of the Immunogenicity Potential for Oligonucleotide-Based Drugs. Nucleic Acid. Ther..

[194] Geary R.S., Norris D., Yu R., Bennett C.F. (2015). Pharmacokinetics, biodistribution and cell uptake of antisense oligonucleotides. Adv. Drug Deliv. Rev..

[195] Roberts T.C., Langer R., Wood M.J.A. (2020). Advances in oligonucleotide drug delivery. Nat. Rev. Drug Discov..

[196] Khalil M., Teunissen C.E., Otto M., Piehl F., Sormani M.P., Gattringer T., Barro C., Kappos L., Comabella M., Fazekas F. (2018). Neurofilaments as biomarkers in neurological disorders. Nat. Rev. Neurol..

[197] Liu Z., Song S.Y. (2025). Genomic and Transcriptomic Approaches Advance the Diagnosis and Prognosis of Neurodegenerative Diseases. Genes.

[198] Bu F.T., Wang H.Y., Xu C., Song K.L., Dai Z., Wang L.T., Ying J., Chen J. (2024). The role of m6A-associated membraneless organelles in the RNA metabolism processes and human diseases. Theranostics.

[199] Faraldi M., Gomarasca M., Sansoni V., Perego S., Banfi G., Lombardi G. (2019). Normalization strategies differently affect circulating miRNA profile associated with the training status. Sci. Rep..

[200] Azam H.M.H., Rossling R.I., Geithe C., Khan M.M., Dinter F., Hanack K., Pruss H., Husse B., Roggenbuck D., Schierack P. (2024). MicroRNA biomarkers as next-generation diagnostic tools for neurodegenerative diseases: A comprehensive review. Front. Mol. Neurosci..

[201] Wilton-Clark H., Yan E., Yokota T. (2024). Preparing for Patient-Customized N-of-1 Antisense Oligonucleotide Therapy to Treat Rare Diseases. Genes.

[202] Alhamadani F., Zhang K., Parikh R., Wu H., Rasmussen T.P., Bahal R., Zhong X.B., Manautou J.E. (2022). Adverse Drug Reactions and Toxicity of the Food and Drug Administration-Approved Antisense Oligonucleotide Drugs. Drug Metab. Dispos..

[203] Kim K.R., Kang J.H., Thai H.B.D., Back J.H., Mao C., Lee J.E., Ko Y.T., Ahn D.R. (2025). Systemic Brain Delivery of Oligonucleotide Therapeutics Enhanced by Protein Corona-Assisted DNA Cubes. Small Methods.

[204] Ji C., Ding L., Jia F., Zhang Z., Long C. (2024). Integrated Transcriptome Analysis Reveals Molecular Subtypes and ceRNA Networks in Multiple Sclerosis. Degener. Neurol. Neuromuscul. Dis..

[205] Joglekar A., Hu W., Zhang B., Narykov O., Diekhans M., Marrocco J., Balacco J., Ndhlovu L.C., Milner T.A., Fedrigo O. (2024). Single-cell long-read sequencing-based mapping reveals specialized splicing patterns in developing and adult mouse and human brain. Nat. Neurosci..

